# Mechanistic principles of an ultra-long bovine CDR reveal strategies for antibody design

**DOI:** 10.1038/s41467-021-27103-z

**Published:** 2021-11-18

**Authors:** Hristo L. Svilenov, Julia Sacherl, Ulrike Protzer, Martin Zacharias, Johannes Buchner

**Affiliations:** 1 Center for Protein Assemblies and Department Chemie, Technische Universität München, 85748 Garching, Germany; 2 Institute of Virology, Technical University of Munich / Helmholtz Zentrum Munich, Munich, Germany; 3 German Center for Infection Research, Munich partner site, Munich, Germany; 4 Center for Protein Assemblies and the Department Physik, Technische Universität München, 85748 Garching, Germany

**Keywords:** Antibodies, Biochemistry, Protein design

## Abstract

Antibodies bind antigens via flexible loops called complementarity-determining regions (CDRs). These are usually 6-20 residues long. However, some bovine antibodies have ultra-long CDRs comprising more than 50 residues organized in a stalk and a disulfide-rich knob. The design features of this structural unit and its influence on antibody stability remained enigmatic. Here, we show that the stalk length is critical for the folding and stability of antibodies with an ultra-long CDR and that the disulfide bonds in the knob do not contribute to stability; they are important for organizing the antigen-binding knob structure. The bovine ultra-long CDR can be integrated into human antibody scaffolds. Furthermore, mini-domains from de novo design can be reformatted as ultra-long CDRs to create unique antibody-based proteins neutralizing SARS-CoV-2 and the Alpha variant of concern with high efficiency. Our findings reveal basic design principles of antibody structure and open new avenues for protein engineering.

## Abstract (web summary)

Certain bovine antibodies have ultra-long long complementarity-determining regions (CDRs) that contain a knob for antigen interaction, which is connected to the antibody through a stalk. Here, the authors combine biophysical experiments and MD simulations and show that the stalk length is critical for the folding and stability of these antibodies. The authors also demonstrate that ultra-long bovine CDRs can be grafted into human antibodies, and furthermore show that de novo designed mini-domains that bind to the SARS-CoV-2 spike protein with high affinity can be integrated as a knob in ultra-long CDRs in bovine and human antibodies, which neutralize SARS-CoV-2.

## Introduction

Human IgG antibodies are glycoproteins composed of two heavy (HC) and two light chains (LCs)^1^. All four chains are covalently connected by disulfide bonds into a characteristic Y-shaped structure. The antigen-binding site is formed by segments in the variable domains of both the heavy (V_H_) and the light chains (V_L_), the so-called complementarity-determining regions (CDRs). These are loops connecting β-strands. V_H_ and V_L_ contribute three CDRs each: CDR-H1, CDR-H2, CDR-H3 for the HC, and CDR-L1, CDR-L2 and CDR-L3 for the LC. In humans, the CDR sequences are rather short and even CDR-H3, which shows the largest variability in length, is typically composed of only 6 to 20 amino acids^2,3^. Longer human CDR-H3s consisting of 25 to 30 residues are also known but they are much less prevalent in comparison to their shorter counterparts^4,5^. As a result of the CDR length, the common human antibodies usually have a large but flat antigen-binding surface that is not suitable for the recognition of some antigens, for example, certain viruses^3,6^.

**Fig. 1:**
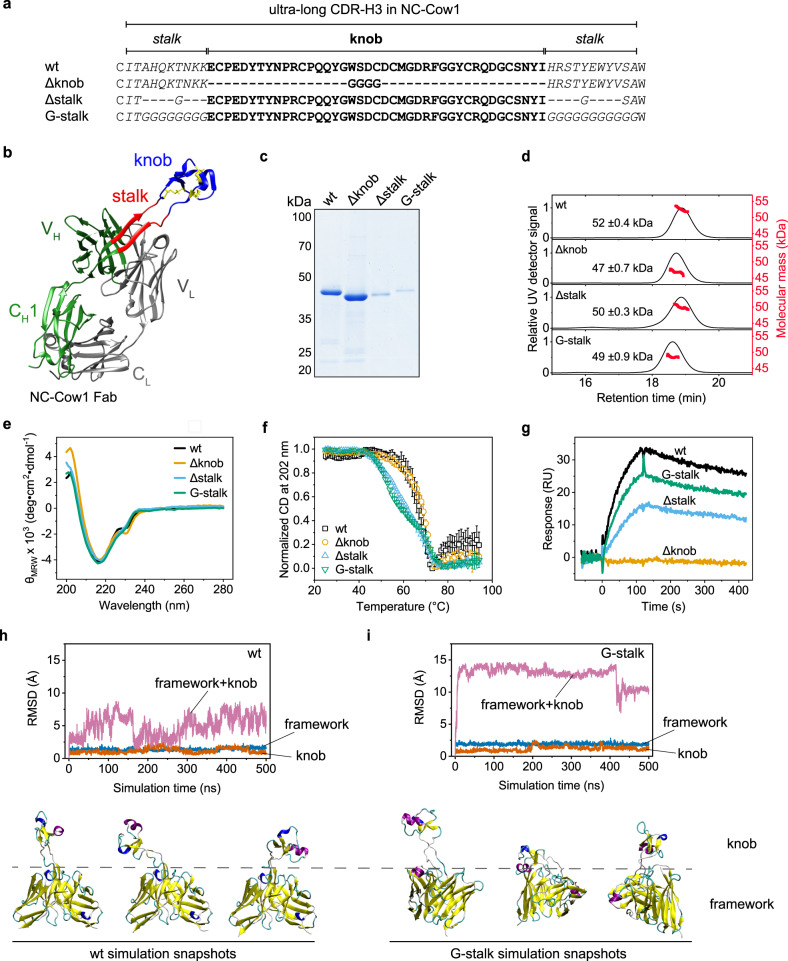
Importance of the stalk and knob in the ultra-long CDR-H3. **a** Sequences of NC-Cow1 mutants with differences in the ultra-long CDR-H3. **b** Crystal structure of NC-Cow1 Fab (PDB:6OO0). **c** Immunoprecipitation of Expi293 supernatants after transient expression of the Fab fragments followed by SDS-PAGE. Two independent experiments. **d** SEC MALS chromatograms of the purified NC-Cow1 Fab variants (the molecular mass is shown in red; the value is mean of triplicates with standard deviation). **e** FUV CD spectra and **f** thermal stability of the NC-Cow1 Fab variants (the points represent the mean of triplicates with standard deviation). **g** Binding of 100 nM NC-Cow1 Fab variants to the immobilized HIV-1 Env protein as determined by SPR. In **e**, **f** and **g**, black is the wildtype (wt) NC-Cow1 Fab, orange is the mutant with deleted knob (Δknob), blue is the mutant with deleted stalk (Δstalk), and green is the mutant with a stalk made of glycine residues (G-stalk). **h** and **i** Comparative MD-simulation on the Fv of NC-Cow1 wt (**h**) and G-stalk (**i**) showing the RMSD deviation (with respect to experimental start structure) of the Fv framework alone (blue), the knob alone (orange), or the motion of the whole structure (knob plus the Fv framework, magenta). Simulation snapshots at different phases of the simulation are indicated.

Antibodies from other species like mouse, chicken, camel, and shark can exhibit different structural features compared to humans^7^. However, despite the structural diversity, antibodies from all species mentioned above have CDRs of a similar average length as the human CDRs^2,3,8,9^. Therefore, it came as a surprise that the CDR-H3s of some bovine antibodies can be extremely expanded^10,11^. These bovine antibodies have the longest naturally occurring CDR sequences known to date. They can consist of 50 to 70 amino acids^12^. Interestingly, the ultra-long CDRs are made of two distinct structures – a β-ribbon “stalk” which protrudes from the HC and a “knob” that sits atop of the “stalk”, giving the entire CDR-H3 a characteristic mushroom shape^13^. The stalk is formed by the sequences at the beginning and at the end of the CDR-H3 insert. What makes this structure even more fascinating is the presence of several disulfide bonds in the “knob”. The above-mentioned features make bovine antibodies with ultra-long CDR-H3 unique binders for antigen epitopes that are less accessible to antibodies from other species^13,14^. This has been demonstrated by immunization of cows with a surface protein of the human immunodeficiency virus (HIV), an HIV-1 Env trimer, and the subsequent isolation of a bovine antibody (NC-Cow1) with broadly neutralizing activity against HIV^15^. Like some other bovine antibodies^13,16,17^, NC-Cow1 has an ultra-long CDR-H3 (60 residues) divided into a stalk and a knob (Fig. 1a, b). The crystal structure of NC-Cow1 bound to its antigen revealed that antigen recognition occurs via the knob^18^. However, the importance of the different structural elements in the ultra-long CDR-H3 for the folding, stability and binding affinity of bovine antibodies remained enigmatic.

Here, we dissected the effects of the structural elements of the ultra-long CDR-H3 on antibody architecture and antigen binding with a view to uncover the potential for engineering new antigen-binding entities. Our study shows that the stalk provides stability and that the disulfide bonds provide conformational constraints required for antigen binding. This information allowed us to create human antibodies with ultra-long CDR-H3s comprising a naturally occurring bovine knob or a target-binding mini-domain from de novo design.

## Results

### The stalk is important for the stability of antibodies with ultra-long CDR-H3s

We were interested to dissect the design principles of naturally occurring ultra-long CDR-H3s. Specifically, we aimed to understand the importance of the stalk and knob regions. We therefore created NC-Cow1 mutants with a knob deletion (Δknob) or a stalk deletion (Δstalk) (Fig. 1a). We also replaced the amino acids in the stalk of NC-Cow1 wildtype (wt) with glycine residues to obtain a mutant with a flexible stalk (G-stalk) (Fig. 1a). The rationale for replacing the stalk with glycine residues was to determine whether the length of the stalk or the specific interactions between the stalk and the framework were important.

We expressed these NC-Cow1 variants transiently as Fab fragments in Expi293 cells. Immunoprecipitation of the respective supernatants showed that the NC-Cow1 wt and the Δknob mutant were secreted well (Fig. 1c); in contrast, the Δstalk and G-stalk mutants were secreted poorly (Fig. 1c). These results suggest that the stalk is a key element for the organization of the ultra-long CDRs and that not only the presence of a stretch of amino acids is important but a stalk with intrinsic structural features conveying stability and a specific three-dimensional structure.

We purified these proteins to homogeneity and determined their structural and functional characteristics. SEC MALS analyses revealed that all four NC-Cow1 Fab variants are heterodimers with the expected molecular mass (Fig. 1d). The secondary structure of the NC-Cow1 mutants investigated was similar to that of the wt as deduced from FUV CD spectroscopy (Fig. 1e). The thermal stability of the constructs was determined by following the change in CD signal with increasing temperature. Interestingly, the Δknob mutant has an almost identical thermal stability (T_M_ = 67.5 °C) compared to NC-Cow1 wt (T_M_ = 65.9 °C), but the Δstalk and G-stalk mutants unfold at lower temperatures with T_M_ values of 56.3 °C and 53.3 °C, respectively (Fig. 1f; Table 1).

**Table 1.** Overview of characteristics of the bovine, human and chimeric Fab variants.

		T_M_ (°C)^a,b^				
	Protein	FUV CD	DSC	nanoDSF	Yield (mg/L)^c^	K_D_ (nM)^d^
NC-Cow1	wt	65.9 ± 1.1	69.4 ± 0.1	70.9 ± 0.0	50	3.2 ± 0.9
	Δknob	67.5 ± 0.6	—	—	—	n.d.
	Δstalk	56.3 ± 0.2	—	—	—	11.0 ± 3.7
	G-stalk	53.3 ± 0.0	—	—	—	4.3 ± 0.6
	(stalk)_2_	63.4 ± 0.7	—	—	—	5.7 ± 1.4
	(stalk)_3_	61.6 ± 1.1	—	—	—	5.3 ± 0.9
	wt→BS1	55.5 ± 0.2	—	—	—	n.d.
	wt→BS2	55.8 ± 0.1	—	—	—	n.d.
	wt→B4	57.9 ± 0.2	—	—	—	n.d.
	wt→B13	55.1 ± 0.2	—	—	—	n.d.
	C(I,IV)S	65.3 ± 0.8	66.8 ± 0.5	—	—	n.d.
	C(III,VI)S	66.9 ± 0.4	67.4 ± 0.7	—	—	n.d.
	C(II,V)S	68.1 ± 0.3	67.9 ± 0.5	—	—	n.d.
	C(I-V)S	66.1 ± 0.8	67.1 ± 0.2	—	—	n.d.
	SB	65.6 ± 0.7	67.0 ± 0.3	—	—	n.d.
	wt→PG16	64.2 ± 0.5	65.3 ± 0.3	—	—	n.d.
	wt→VRC26.25	64.8 ± 0.4	67.9 ± 0.1	—	—	n.d.
	+LCB1	—	66.4 ± 0.0	68.6 ± 0.0	45	0.6 ± 0.1
trastuzumab	wt	—	81.2 ± 0.1	81.6 ± 0.0	20	n.d.
	+6 bovine CDRs	—	—	71.7 ± 0.0	1	3.0 ± 0.4
	+knob	—	78.3 ± 0.0	78.8 ± 0.0	20	17.6 ± 5.5
	+LCB1	—	77.7 ± 0.0	78.4 ± 0.1	13	0.9 ± 0.1
32H+109L	wt	—	76.8 ± 0.1	77.6 ± 0.0	20	n.d.
	+6 bovine CDRs	—	63.8 ± 0.1	63.0 ± 0.0	35	4.9 ± 0.3
	+knob	—	—	78.0 ± 0.0	2	6.5 ± 1.0
	+LCB1	—	—	76.4 ± 0.1	3.5	0.5 ± 0.1
PGT145	wt	—	—	75.5 ± 0.1	20	n.d.
	+6 bovine CDRs	—	—	73.4 ± 0.1	2	4.0 ± 0.4
	+knob	—	—	75.5 ± 0.6	5.5	7.7 ± 3.3
	+LCB1	—	-	74.3 ± 0.1	5.0	0.5 ± 0.1

^a^The FUV CD and nanoDSF values are mean of triplicates except for wt→PG16 and wt→VRC26.25 (duplicates), the DSC values are mean of at least duplicates. The error is the standard deviation.

^b^Several proteins had a second unfolding transition. Δstalk T_M_2 = 69.4 ± 0.7 °C; G-stalk T_M_2 = 70.4 ± 1.4 °C; wt→BS1 T_M_2 = 69.4 ± 1.5 °C; wt→BS2 T_M_2 = 67.7 ± 1.6 °C; wt→B4 T_M_2 = 73.0 ± 1.0 °C; 32H+109L + 6 bovine CDRs T_M_2 = 76.0 ± 0.2 °C.

^c^The relative yields are given only for Fabs where the yields were obtained by expressing the proteins in parallel using the same batch of Expi293 cells. The values represent approximate amount of protein that was obtained after all purification steps. For other constructs, immunoprecipitation was used for comparisons.

^d^The antigen is the HIV-1 Env protein except for the variants with LCB1 peptide where the antigen is the RBD from SARS-CoV-2. n.d. - not determined/no binding. The K_D_ values are mean of triplicates with standard deviation.

To test how the architecture of the ultra-long CDR affects antigen binding, the wt and variants were assayed concerning their affinity for the HIV-1 Env protein by SPR. As expected, the Δknob mutant did not bind to the antigen with detectable affinity (Fig. 1g). Interestingly, the NC-Cow1 mutants without a stalk or with a stalk made of glycines bound to the HIV-1 Env protein. The affinity constants determined for the constructs (Supplementary Fig. 1) revealed that NC-Cow1 wt binds with a K_D_ = 3.2 nM; the G-stalk mutant has a similar K_D_ of 4.3 nM, while the Δstalk mutant showed only a slightly weaker binding and a K_D_ = 11 nM (Table 1).

Molecular dynamics (MD) simulations on the Fv of NC-Cow1 provided insight into the importance of the stalk. The mutant with deleted stalk could not be modeled due to severe steric clashes between the knob and the Fv framework when the stalk was removed, which corresponds to the lower stability and antigen-binding affinity of the Δstalk mutant compared to wt. MD simulations on the G-stalk mutant indicate that replacing the stalk with Gly residues strongly increases the motion of the knob relative to the Fv framework (magenta lines in Fig. 1h and i); however, the root-mean-square deviation (RMSD) of the knob itself with respect to the start knob structure is remarkably similar between wt and G-stalk (orange lines in Fig. 1h, i). Hence, the knob retains its rigidity when the stalk is highly flexible, which explains the preserved antigen binding by the G-stalk mutant. The RMSD of the core part of the G-stalk (blue lines in Fig. 1h and i) is on average slightly larger (<RMSD> ~2.2 Å) than the corresponding mean deviation for the wt (<RMSD> ~1.5 Å). Hence, the highly flexible stalk disturbs the Fv framework structure of NC-Cow1 which agrees with the lower thermal stability of G-stalk compared to wt.

Taken together, the data reveals that the specific stalk structure in NC-Cow1 wt is important for the overall stability but less for the folding of the knob.

### Stalk extensions in the bovine ultra-long CDR-H3 are not beneficial

Knowing that stalk deletions have detrimental effects on the stability of NC-Cow1, we asked how stalk extensions will affect the antibody. Parts of the two strands of the stalk in NC-Cow1 form a β-ribbon (Fig. 1b)^18^. We were interested whether this naturally-occurring stalk could be extended by replicating the rigid β-ribbon structure. We therefore repeated these complementary residues to obtain mutants with a double and a triple stalk, named (stalk)_2_ and (stalk)_3,_ respectively (Supplementary Fig. 2a). We observed that extending the stalk in this way reduced the secretion levels of the corresponding Fab fragments from mammalian cells (Supplementary Fig. 2b). The secondary structures of the purified variants remained unchanged (Supplementary Fig. 2c), but the thermal stability of the Fabs with longer stalks was lower compared to the wt (Table 1 and Supplementary Fig. 2d). The (stalk)_2_ and (stalk)_3_ mutants bound to the antigen with K_D_s of 5.7 nM and 5.3 nM, respectively (Table 1 and Supplementary Fig. 2e and f).

Taken together, the stalk deletions and extensions show that while both shortening and elongating the stalk is possible, it is not beneficial. Thus the stalk length in NC-Cow1 wt has evolved for optimal folding, stability and binding to the antigen.

### Replacing the ultra-long CDR-H3 with short bovine CDRs reduces Fab stability

Bovine antibodies like NC-Cow1 have the longest CDR-H3s found in nature. One could presume that such long CDRs are avoided by evolution because they could impose constraints on folding and stability. However, despite the complexity of its CDR-H3, the NC-Cow1 Fab was well secreted (Fig. 1c) and thermally stable (Fig. 1f). This raises the question whether the framework in these bovine antibodies has exceptional stability that can compensate for potential destabilization induced by the ultra-long CDR-H3. To test this hypothesis, we replaced the ultra-long CDR-H3 in NC-Cow1 with short bovine CDR-H3 loops from the bovine antibodies BS1, BS2, B4 and B13 (Supplementary Fig. 3a)^13,19^. However, in contrast to our assumption, the mutants with short bovine CDR-H3s were secreted poorly compared to the NC-Cow1 wt (Supplementary Fig. 3b). In line with the potential folding problems in the cell, the grafted variants with short bovine CDR-H3s affected the secondary structure of the Fabs (Supplementary Fig. 3c) and reduced their thermal stabilities (Table 1 and Supplementary Fig. 3d). We wondered whether the high stability is a specific feature of the cow Fabs with ultra-long CDRs and therefore also characterized bovine Fabs which naturally contain short CDR-H3s, namely B4 and B13. We found that they formed heterodimers (Supplementary Fig. 3e), had the characteristic secondary structures (Supplementary Fig. 3f), and high thermal stability (Supplementary Fig. 3g).

Thus, the bovine antibodies with ultra-long CDR-H3s evolved to maintain the typical stability of bovine antibodies and are in this respect indiscriminable from their natural counterparts with short CDR-H3s. Surprisingly, introducing short loops instead of the ultra-long CDR-H3 in the framework is detrimental for stability.

### The disulfide bonds rigidify the knob to allow antigen binding

**Fig. 2:**
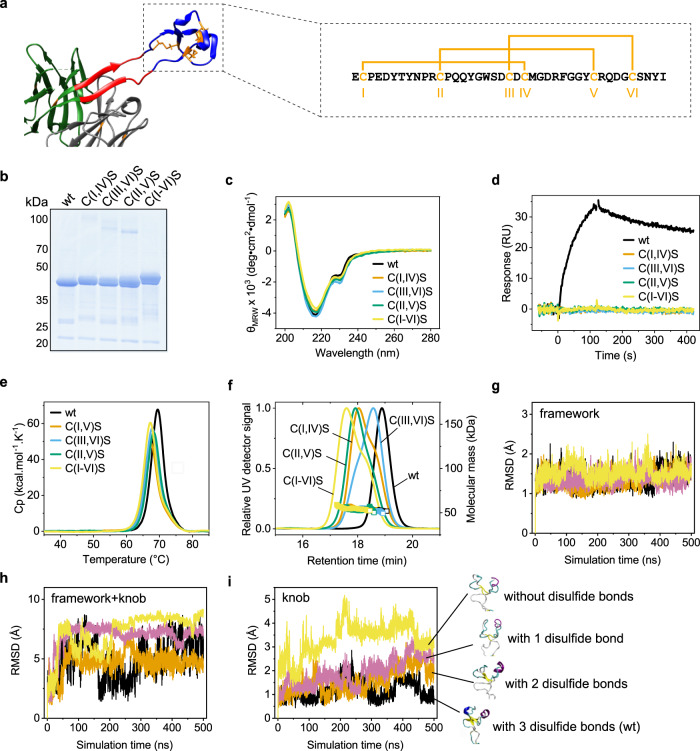
Role of the disulfide bonds in the knob. **a** Cysteines and disulfide bond pattern in the knob NC-Cow1 (PDB:6OO0). **b** Immunoprecipitation of Expi293 supernatants after transient expression of Fab fragments followed by SDS-PAGE. Two independent experiments. **c** FUV CD spectra of the NC-Cow1 Fab wt compared to C → S mutants. **d** Binding of 100 nM NC-Cow1 Fab variants to immobilized HIV-1 Env protein in SPR. **e** Thermal unfolding of the NC-Cow1 Fab wt and mutants measured with DSC. **f** Molecular mass and eluting peaks of NC-Cow1 Fab wt and C → S mutants in SEC MALS. In **c**, **d**, **e** and **f**, black is the wildtype (wt) NC-Cow1 Fab, the remaining colors represent different NC-Cow1 Fab mutants with cysteine residues replaced by serine residues in the knob. Refer to **a** for the exact position of the mutations. **g**, **h** and **i** Comparative MD simulations of the Fv of NC-Cow1 wt (black) and variants with two (orange), one (magenta) or no (yellow) disulfide bonds in the knob. RMSD for the **g** framework, **h** the whole structure (knob plus the framework) and **i** the knob itself. A snapshot of the finally sampled knob structure is indicated.

The bovine ultra-long CDR-H3s contain several cysteines that form disulfide bonds^13^; NC-Cow1 has three disulfide bonds that form a characteristic pattern in the knob (Fig. 2a)^18^. Alanine scanning had indicated that some of these cysteines (Cys II and Cys V in Fig. 2a) are crucial for binding to the antigen^18^. However, little is known of the importance of each disulfide bond for NC-Cow1 stability and antigen binding.

To study the importance of disulfide bonds in the knob of NC-Cow1, we created mutants where each pair of cysteines that forms a disulfide bond (Fig. 2a) was replaced with a pair of serines, (C(I,IV)S, C(III,VI)S, and C(II,V)S). In addition, we created a mutant, C(I-V)S, where all six cysteines in the knob were replaced by serine residues. All C → S mutants were secreted at levels comparable to those of the NC-Cow1 wt Fab in mammalian cells (Fig. 2b) and exhibited similar CD spectra (Fig. 2c); remarkably, however, all mutants had lost their antigen-binding properties (Fig. 2d). The question remaining was whether the disulfide bond deletions in the knob affect the overall stability of NC-Cow1 Fab. Preliminary experiments monitoring the thermal stability of the C → S mutants by FUV CD spectroscopy revealed transitions similar to those observed for NC-Cow1 wt (Supplementary Fig. 4a). To determine whether there are subtle changes in the thermal unfolding that were not detected by FUV CD, we used differential scanning calorimetry (DSC). NC-Cow1 wt Fab exhibited one unfolding peak in the thermogram with a T_M_ of 69.4 °C (Fig. 2e) that corresponds well to the observed transition in FUV CD (Fig. 1f). The mutants with pairwise C → S substitutions also showed only one unfolding transition and had only slightly lower T_M_ values (Fig. 2e and Table 1). Noteworthy, even the C(I-V)S mutant that cannot form any disulfide bonds in the knob exhibits a thermal stability very similar to the NC-Cow1 wt with a T_M_ of 67.1 °C.

Since antigen binding is abolished in the cysteine mutants, we hypothesized that removing disulfide bonds from the knob will cause structural changes that could affect not only the interaction with the antigen but also the hydrodynamic properties of NC-Cow1 Fab. To test this, we performed SEC MALS experiments and observed that all C → S mutants exist as heterodimers with the correct molecular mass (Fig. 2f); however, the mutants with replaced pairs of cysteines eluted earlier compared to the wt and showed asymmetric peaks indicating conformational heterogeneity (Fig. 2f). The C(I-V)S mutant with no cysteines in the knob showed the shortest retention time (Fig. 2f).

We then used MD simulations to explain the effects of disulfide bond removal on antigen binding and stability. Comparative MD simulations on the Fv of the NC-Cow1 wt and the cysteine variants indicate that the framework is not affected as no deviation (RMSD) was observed when disulfide bonds are removed from the knob (Fig. 2g). This result is in line with the observed small effect of the disulfide bond removal on protein stability. Also, the overall motion of framework plus knob is only modestly increased upon disulfide bond removal (Fig. 2h). However, the RMSD of the knob itself increased significantly over time already upon the removal of one disulfide and even larger deviations relative to the starting structure were observed when removing all disulfide bonds (Fig. 2i). These structural changes in the knob of the ultra-long CDR-H3 in the absence of disulfide bonds explains the experimentally observed loss of antigen binding of these variants.

Because the formation of multiple disulfide bonds within one structural element can be challenging, we wondered whether a salt bridge can be introduced to replace a disulfide bond. To test this, we created a mutant where we replaced Cys I and Cys IV (Fig. 2a) with Arg and Glu respectively to create a mutant called NC-Cow1 SB. This mutant was well secreted but did not bind to the antigen (Supplementary Fig. 4).

Overall, the C → S mutants of NC-Cow1 revealed important information. The disulfide bonds in the knob are crucial for antigen binding by imposing conformational restraints in the paratope; however, the disulfide bonds in the knob do not contribute significantly to the overall Fab stability - even when disulfide bonds are absent from the knob of NC-Cow1. From a stability perspective, our results show that NC-Cow1 can tolerate not only folded and rigid knobs in its ultra-long CDR-H3 but also a large, disordered domain.

### The bovine knob can be replaced by long human CDRs

It had previously been shown that the knob in bovine antibodies with ultra-long CDR-H3 can be replaced by short peptides^20,21^ or small cytokines^22,23^ to create functional fusion proteins. Since the NC-Cow1 Fab remained stable after we removed all disulfide bonds in the knob, we wondered whether the structure would tolerate the replacement of the knob with non-bovine long CDRs. In fact, there are several human antibodies that have a long CDR-H3 that folds into a hammerhead structure (e.g. the PG16 antibody)^24^ or contains a beta strand (e.g. the VRC26.25 antibody)^25^ that are important for binding to the antigen. The CDR-H3 of PG16 does not contain cysteines, while the CDR-H3 of VRC26.25 has one disulfide bond. We were therefore interested if the grafting of these two different human CDR structures will affect the stability of NC-Cow1. Moreover, we wanted to test whether the grafted human CDR-H3 will still exhibit binding to the antigen. We therefore grafted sequences from the CDR-H3 of PG16 and VRC26.25 in the place of the knob in NC-Cow1 to create the wt→PG16 and wt→VRC26.25 mutants (Supplementary Fig. 5a). These NC-Cow1 Fab mutants were well secreted (Supplementary Fig. 5b) and had wildtype-like secondary structure (Supplementary Fig. 5c). The thermal stability of the mutants with replaced knobs was only slightly lower (Table 1 and Supplementary Fig. 5d); wt→PG16 has a T_M_ of 65.3 °C, the T_M_ of wt→VRC26.25 is 67.9 °C. Like the C → S mutants in the previous section, wt→PG16 and wt→VRC26.25 showed differences in the peak shape and elution time in SEC MALS (Supplementary Fig. 5e) and did not bind to the antigen (Supplementary Fig. 5f).

All in all, it seems that the human CDR-H3 sequences can replace the knob of NC-Cow1 with only minor effects on folding and thermal stability. These findings suggest that the landscape of possible donor sequences that can be grafted onto ultra-long CDR-H3s is not limited to short peptides and compactly folded proteins. However, the grafted sequences need to contain the entire antigen-binding properties, like the bovine knob, to obtain functional antibodies.

### Bovine antibodies with ultra-long CDR-H3 can be humanized by two approaches

NC-Cow1 broadly neutralizes HIV and presents an attractive option for the prevention and therapy of acquired immunodeficiency syndrome^15^. For clinical success, animal antibodies need to be humanized to reduce immunogenicity risks^26^. The humanization of bovine antibodies with ultra-long CDR-H3s has not been demonstrated yet. It remained unclear whether human antibody scaffolds can tolerate the ultra-long bovine CDR-H3 without detrimental effects on folding, stability, and antigen binding.

Humanization has been achieved by replacing the CDRs in a human scaffold with the CDRs from an animal antibody^27^. This approach often requires the grafting of several or all 6 CDRs that form the antigen-binding site. In bovine antibodies with ultra-long CDR-H3, the antigen-binding site is formed by the knob; however, it was suggested that successful humanization might require grafting of the remaining CDRs that could provide structural support for the ultra-long CDR-H3^7^.

**Fig. 3:**
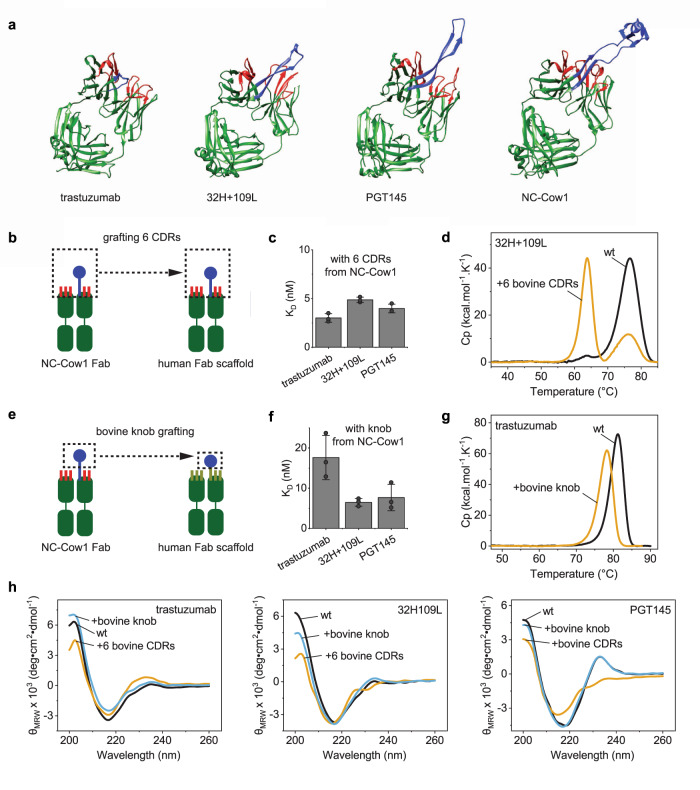
Humanization approaches for bovine antibodies with ultra-long CDR-H3s. **a** A comparison between the 3D structures of human Fab scaffolds (trastuzumab (PDB: 6B9Z), 32H+109L (PDB: 5CEX), PGT145 (PDB: 3U1S)) and NC-Cow1 Fab (PDB:6OO0). The CDR-H3 is colored in blue, the remaining CDRs are in red. **b** Humanization by transferring all 6 CDRs from NC-Cow1 to a human scaffold. The ultra-long cow CDR-H3 is in blue. The remaining five cow CDRs are in red. The frameworks are in dark green. **c** Binding affinity of human Fabs with 6 bovine CDRs to the HIV-1 Env antigen in SPR (mean of triplicates with standard deviation). **d** Thermal stability of 32H+109L with 6 cow CDRs compared to the parent human Fab. **e** Humanization by inserting the knob from NC-Cow1 in the tip of CDR-H3 of a human scaffold. The bovine knob is in blue. The remaining five cow CDRs are in red. The human CDRs are in light green. The frameworks are in dark green. **f** Binding affinity of human Fabs with bovine knob insertions in CDR-H3 (mean of triplicates with standard deviation). **g** Thermal stability of trastuzumab with a knob insertion compared to the parent Fab. **h** FUV CD spectra of human and chimeric Fabs. Sequence alignments of the wt proteins and corresponding mutants can be found in supplementary data (Supplementary Fig 13). In **g** and **h**, black is the wildtype (wt) protein, blue is a mutant with a bovine knob grafted onto a human CDR-H3, orange is a mutant where all six human CDRs are exchanged for all six bovine CDRs.

To test whether the humanization of bovine antibodies with ultra-long CDR-H3 is possible, we tested three different human scaffolds (Fig. 3a). First, we selected an intrinsically stable Fab scaffold (trastuzumab) that has already been used in humanization^28^ and maintained excellent stability^29^. Second, we used BLAST^30^ to compare bovine and human antibody sequences in the PDB. We identified several human Fd sequences with high homology to bovine antibodies (Supplementary Fig. 6) and subsequently the corresponding human and bovine LCs (Supplementary Fig. 7). We selected the human Fab 32H+109L^31^ that has overall high homology to the bovine Fab fragments with ultra-long CDR-H3s as a human scaffold for our experiments. Third, we included a human Fab (PGT145)^32^ with a long (31 residue) CDR-H3. The sequence alignments of these proteins and the corresponding mutants can be found in supplementary data.

We replaced the CDRs in the human Fabs with all 6 CDRs from NC-Cow1 (Fig. 3b). The CDR grafting reduced the production yields of the trastuzumab Fab and PGT145 Fab 20 times and 10 times, respectively (Table 1). Interestingly, the yields of 32H+109L were not negatively affected by the CDR grafting. All three human scaffolds with CDRs from NC-Cow1 bound to the HIV-1 Env antigen with K_D_s between 3 nM and 4.9 nM (Table 1 and Fig. 3c and Supplementary Fig. 8a). Due to low yields of some purified proteins, we used fluorescence spectroscopy (nanoDSF) to study the thermal stability of the chimeric constructs and observed that all chimeric Fabs were less stable compared to the wt proteins (Table 1 and Supplementary Fig. 8d). DSC analysis of the best acceptor (32H+109L) of the cow CDRs (Fig. 3d) confirmed the T_M_s determined by the fluorescence-based approach (Table 1).

To reduce the size of the transferred structural element, we inserted only the knob (without the stalk) of NC-Cow1 into the tip of human CDR-H3s (Fig. 3e). Overall, the knob grafting had less negative impact on folding and stability compared to grafting all CDRs; the production yield of trastuzumab+knob was the same as observed for the wt, while the yields of 32H+109L + knob and PGT145 + knob were around 10 and 4 times lower compared to the wt (Table 1).

The K_D_ of trastuzumab+knob to the HIV-1 Env protein (Fig. 3f and Supplementary Fig. 8b) was 17.6 nM (Table 1); this was the weakest affinity of all chimeric constructs tested and similar to the value obtained for the Δstalk mutant of NC-Cow1. This suggests that in both trastuzumab+knob and NC-Cow1 Δstalk, the knob is located close to the V_H_ framework, which has negative effects on antigen binding. The 32H+109L + knob and PGT145 + knob had K_D_s of 6.5 nM and 7.7 nM, respectively (Table 1); the CDR-H3s of these two human Fabs are longer which positions the inserted knob further away from the V_H_ framework in comparison to trastuzumab. In addition to the SPR measurements, we tested whether the human Fabs with grafted bovine knobs bind to the soluble HIV Env trimer (Supplementary Fig. 8c). All three Fabs bound the trimeric HIV Env as seen by the molecular weight shift in SEC-MALS. It turned out that 32H+109L + knob and PGT145 + knob are better binders than trastuzumab + knob which supports our SPR data. In agreement with the results shown above, the optimal distance between the V_H_ and the knob seems to be important.

Knob grafting had less detrimental effects on the thermal stability of the Fabs compared to grafting all 6 CDRs (Table 1 and Supplementary Fig. 8d); correspondingly, the secondary structure of the human Fabs with grafted knobs was more similar to the parent molecule than the human Fabs with 6 grafted bovine CDRs (Fig. 3h). We were also interested whether the knob grafting results in more aggregation-prone molecules compared to the parent antibodies. We therefore used SEC to analyze purified wt and knob mutant pairs (Supplementary Fig. 9a). There were only slightly more aggregates detected in the chimeric proteins and none of the Fabs aggregated during incubation at 50 °C for 24 h (Supplementary Fig. 9a). Since knob grafting is an unconventional humanization approach, we also wanted to test whether we can produce 32H+109L + knob and PGT145 + knob as full-length IgGs. We therefore fused the Fd sequences to the Fc from trastuzumab to obtain HCs and produced the IgGs in mammalian cells. We purified the IgGs secreted in the cell supernatant by protein A chromatography and analyzed the proteins with SEC MALS (Supplementary Fig. 9b). The chimeric IgGs with a grafted bovine knob showed one main peak with a molecular mass around 150 kDa and contained only small fractions of aggregates and fragments (Supplementary Fig. 9b).

Further, we were interested whether the motion of the bovine knob relative to the Fv framework is preserved upon grafting the bovine CDRs or only the bovine knob onto human antibodies. We therefore performed MD simulations with PGT145 wt, PGT145 with all 6 CDRs from NC-Cow1, and with PGT145 + knob (Supplementary Fig. 10). We observed that the overall knob dynamics in the chimeric structures was similar to that of the NC-Cow1 wt. Thus, it can be expected that also the positioning of the knob and its structural isolation in the chimeric constructs with a grafted bovine knob is similar to NC-Cow1 wt.

Overall, both humanization approaches (CDR grafting or knob grafting) seem to work for the bovine NC-Cow1. In the case of CDR grafting, the human scaffold with the highest homology to the bovine sequences (32H+109L) proved to be the best acceptor yielding a well-secreted chimeric protein combining excellent antigen binding with favorable biophysical properties. In the case of knob grafting, a human Fab with a longer CDR-H3 should be used for optimal antigen binding.

### Antigen-binding peptides from de novo design can be grafted as ultra-long CDRs

As demonstrated above, each of the disulfide bonds in the knob is crucial for antigen binding. This restricts approaches for the screening of display libraries to obtain binders; e.g. just 17% of a mammalian library with cysteine-rich peptides was shown to be properly folded^33^. Thus, it would be advantageous to combine the stalk concept with different structural elements displaying the desired antigen-binding properties which are ideally free of cysteines.

**Fig. 4:**
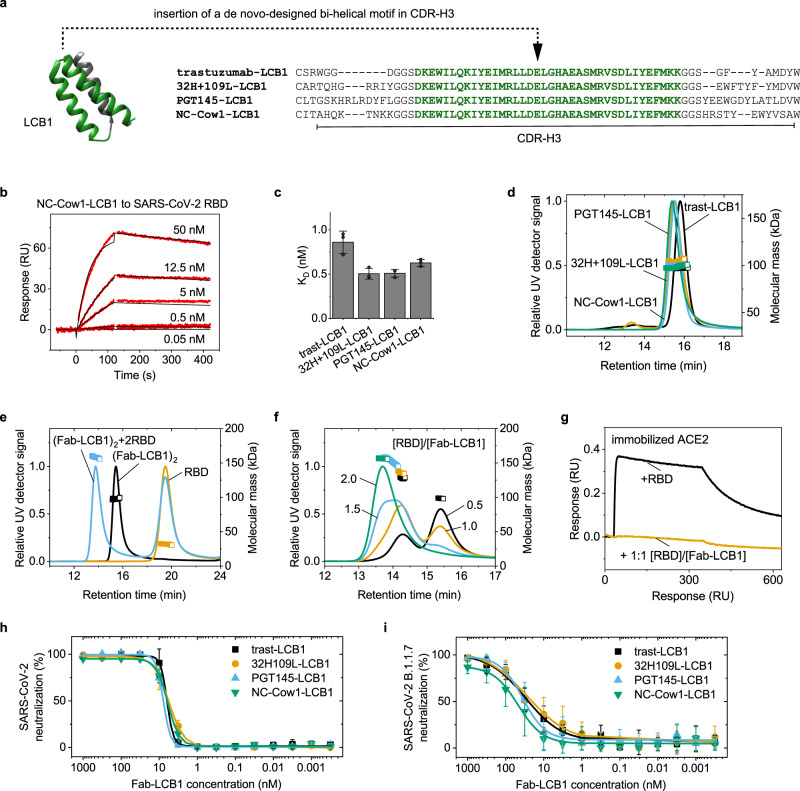
Grafting of de novo designed mini-domains against SARS-CoV-2 into CDR-H3 of bovine and human Fab fragments. **a** Structure of tri-helical LCB1 peptide (PDB: 7JZU) discovered by de novo design. The two helices (green) that bind to SARS-CoV-2 spike were inserted in the CDR-H3s of bovine and human antibodies. **b** Binding of NC-Cow1 with LCB1 insertion to the SARS-CoV-2 RBD in SPR. **c** Affinity constants of the four Fab-LCB1 proteins to SARS-CoV-2 RBD measured with SPR. Mean values of triplicates with standard deviations. **d** The Fab-LCB1s form dimers with a molecular mass around 100 kDa showing that the bi-helical insertion from LCB1 is not only an antigen-binding site but also a dimerization motif. **e** SEC MALS shows that the NC-Cow1-LCB1 bound two RBDs, when the RBD is in 5x molar excess. The NC-Cow1-LCB1 alone is in black, the RBD alone in yellow, the mixture of both is in blue. **f** Substoichiometric concentrations of the RBD lead to the formation of complexes with NC-Cow1-LCB1 that contain one or two RBDs. The following RBD/NC-Cow1-LCB1 ratios are depicted – 0.5 (black), 1.0 (yellow), 1.5 (blue), 2.0 (green). **g** The SARS-CoV-2 RBD cannot bind to immobilized ACE2 receptor when the RBD is premixed 1:1 with NC-Cow1-LCB1. **h** Neutralization of infectious SARS-CoV-2 by trast-LCB1 (IC50 6.3 nM), 32H+109L-LCB1 (IC50 5.7 nM), PGT145-LCB1 (IC50 7.9 nM) and NC-Cow1-LCB1 (IC50 6.1 nM). **i** Neutralization of infectious SARS-CoV-2 Alpha (B.1.1.7) by trast-LCB1 (IC50 30 nM), 32H+109L-LCB1 (IC50 27 nM), PGT145-LCB1 (IC50 35 nM) and NC-Cow1-LCB1 (IC50 47 nM). Data given are means from six curves ±SEM from six independent experiments. In **d**, **h** and **i**, black is trast-LCB1, orange is 32H+109L-LCB1, blue is PGT145-LCB1, green is NC-Cow1-LCB1.

Recently, cysteine-free tri-helical peptides that bind with picomolar affinity to the spike protein of SARS-CoV-2 have been identified by de novo design^34^. One of the most potent peptides (LCB1) isolated by this approach interacts with the receptor binding domain (RBD) of SARS-CoV-2 through two alpha helices (marked in green in Fig. 4a). We hypothesized that we could use these helices in the context of an ultra-long CDR-H3. To test this, we inserted the sequence encoding the two helices into the CDR-H3 of three human and one bovine Fab fragment (Fig. 4a). All four Fab fragments with the LCB1 insertion were correctly folded (Supplementary Fig. 11a) and bound with sub-nanomolar affinity to the RBD of SARS-CoV-2 (Fig. 4b, c and Supplementary Fig. 11b). Remarkably, the Fab-LCB1s formed complexes with molecular masses corresponding to that of two Fab fragments (Fig. 4d). Thus, the bi-helical insertion from LCB1 seems to provide not only a high-affinity binding site for SARS-CoV-2, but also a dimerization motif.

We then used SEC MALS to determine the stoichiometry of the complex between SARS-CoV-2 RBD and a dimeric Fab NC-Cow1-LCB1. When mixing the Fab with a molar excess (5x) of the RBD, the Fab-LCB1 complex had the molecular weight of NC-Cow1-LCB1 bound to two RBDs (Fig. 4e). Thus, one dimeric Fab-LCB1 can bind two RBDs.

We also tested sub stoichiometric ratios between the RBD and NC-Cow1-LCB1 (Fig. 4f). When the [RBD]/[NC-Cow1-LCB1] ratio was 0.5 or 1.0, mostly a complex with only one RBD was formed; at a ratio of 1.5, both complexes with one and two RBD bound to the NC-Cow1-LCB1 were observed; at a ratio of 2.0, the dominant species was NC-Cow1-LCB1 bound to two RBDs.

An important question for therapeutic application is whether the engineered Fabs can block the interaction of the SARS-CoV-2 RBD with the human ACE2 protein. The RBD alone binds efficiently to the immobilized ACE2 receptor in biolayer interferometry (Fig. 4g). When the RBD is premixed 1:1 with NC-Cow1-LCB1, the binding to ACE2 is abolished. Thus, the Fabs with LCB1 peptide insertion block the interaction between the RBD and the ACE2 receptor.

To test whether the constructs neutralize infectious virus and prevent cell infection, we determined the neutralization efficiency of the four Fabs with LCB1 insertions for SARS-CoV-2, isolated early in the pandemic in January 2020, and the SARS-CoV-2 Alpha (B.1.1.7) variant of concern which is currently one of the most prevalent variants worldwide. All four proteins neutralized SARS-CoV-2 very potently with 50% inhibitory concentrations (IC50) between 5.7 nM and 7.9 nM (Fig. 4h). The Alpha variant was also neutralized efficiently with IC50 values between 27 nM and 47 nM.

Interestingly, the Fab-LCB1 proteins have similar thermal stabilities as the wt Fabs and the Fabs with a bovine knob (Table 1 and Supplementary Fig. 11c). This is remarkable since the structure of the mini-domain from LCB1 and the bovine knob are completely different. It reveals that the same Fab scaffold can equally well tolerate the insertion of different naturally occurring and de novo designed structures in the CDR-H3. This finding reveals a new strategy for the rapid discovery of highly potent antibodies for biomedical applications.

## Discussion

The discovery of bovine ultra-long CDRs together with the recent crystal structure of NC-Cow1^18^ was a paradigm shift concerning the conformational space available for paratope structures of antibodies.

Our analysis revealed that the bovine knob acts as an individual entity that has little effect on the secretion and stability of NC-Cow1. The Δknob mutant behaved like the wt protein with one major difference – the complete loss of antigen binding. We were interested if other bovine knobs also do not influence antibody stability. We therefore expressed and tested the Fab of BOV-7, another bovine antibody with an ultra-long CDR-H3. Strikingly, the removal of the knob (46 residues) from BOV-7 also did not affect the secretion by mammalian cells and the stability of the molecule (Supplementary Fig. 12). This further supports the hypothesis that the knobs have evolved as antigen-binding entities that are isolated structurally and concerning stability from the remainder of the antibody.

**Fig. 5:**
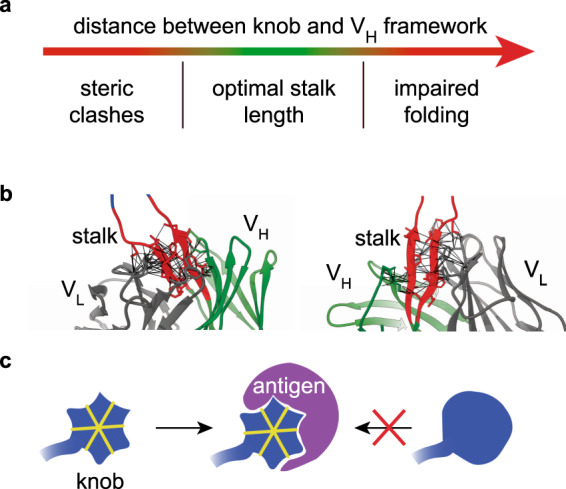
Principles learned from bovine antibodies with ultra-long CDRs. **a**. An optimal stalk length in the ultra-long CDR-H3 ensures proper folding without steric clashes between the knob and residues in the variable domains. **b** There is a consensus between the stalk of CDR-H3 with some of the other CDRs. Depicted are part of the variable domains of NC-Cow1 (PDB:6OO0). The bovine stalk in CDR-H3 is in red, the remaining of the V_H_ domain is in green, the V_L_ domain is in gray. The black lines illustrate a network of potential contacts between the residues in the stalk and some of the other CDRs. **c** The disulfide bonds (yellow lines) in the ultra-long CDR-H3 (blue) provide conformational restraints crucial for binding to the antigen (magenta).

In contrast to the role of the knob, our results highlight the importance of the stalk in the ultra-long CDR-H3 for the folding and stability of the respective antibody (Fig. 5a). Removing the stalk creates structural clashes between the knob and the antibody framework as manifested in the poor folding in vivo and lower thermal stability. Prolonging the stalk negatively affected folding in vivo. It therefore seems that an optimal stalk length evolved during the development of the ultra-long CDRs. Interestingly, the distance between the knob and the framework is conserved among different bovine antibodies^13,16–18^.

Obviously, the evolution of a stable stalk structure allowed isolating the effects of inserting an additional domain between two strands into the beta barrel of the immunoglobulin fold. In agreement with this notion, NC-Cow1 exhibited an intrinsic stability similar to two bovine antibodies with short CDR-H3s. Surprisingly, the replacement of the ultra-long bovine CDR-H3 with a short bovine CDR-H3s negatively affected the in vivo folding and stability of NC-Cow1. A likely explanation is that the ultra-long CDR-H3 exhibits stabilizing interactions with the other CDRs in the V_H_ and V_L_ domains. Potentially stabilizing interactions between the stalk and the other CDRs can be seen in the crystal structure of NC-Cow1^18^ (Fig. 5b) and other bovine antibodies^13,16,17^. The negative consequences of replacing stalk residues by glycines fits the picture. In this case, the MD simulations indicate a strongly enhanced mobility of the knob relative to the framework but little effect on the knob structure itself. This reveals that the relative geometrical arrangement of the knob and framework affect stability but are not critical for antigen binding. For engineering antibodies with ultra-long CDRs, the specific interactions between amino acids in the stalk and other CDRs are an important aspect to consider. In this context, it would be important to establish screening technologies for antibodies with ultra-long CDR-H3s. The fact that the conformational restraints by disulfide bonds in the knob are crucial for antigen binding of NC-Cow1 however presents a challenge for the development of screening libraries using phage, yeast or mammalian display libraries (Fig. 5c)^35^. Recent work has demonstrated that disulfide-rich peptides can in principle be isolated using mammalian display screening. However, correct folding on the cell surface remains a challenge^33^.

**Fig. 6:**
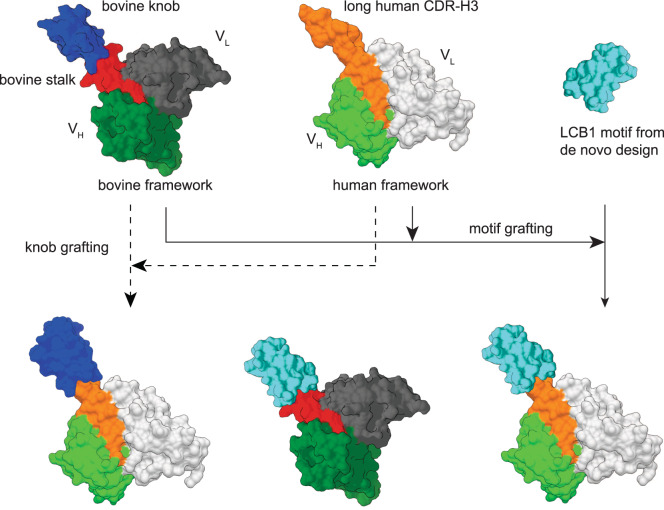
Design principles for the development of bovine and human antibodies with ultra-long CDRs. Naturally occurring bovine knob can be grafted onto a human antibody scaffold. Peptide motif from de novo design can be grafted either onto bovine or human antibodies. The colors depict the following – bovine knob (dark blue), bovine stalk (red), bovine V_H_ except CDR-H3 (dark green), bovine V_L_ (dark gray), human CDR-H3 (orange), human V_H_ except CDR-H3 (light green), human V_L_ (light gray), motif from de novo peptide LCB1 (light blue).

The humanization of antibodies obtained by animal immunization is important to reduce immunogenicity risks in humans^36^. In this study, we identified a human scaffold (32H+109L) which is an excellent receptor for the grafting of the CDRs from NC-Cow1. The chimera did not impact folding in vivo, but a reduction in thermal stability was observed pointing to additional stabilizing interactions between the CDRs and framework in the authentic context which are not present in the chimera. It is also possible that the relative orientation of the grafted bovine CDRs is not maintained upon grafting, which might explain the reduced thermal stability. Additional mutations in the Vernier zone of the antibody framework could be a feasible approach to solve this issue^37^. We also show that this problem can be circumvented by knob grafting (Fig. 6). This strategy is less invasive with the best results obtained for a bovine knob grafted on the tip of a long human CDR-H3; in line with our notion that a stalk is required to isolate the knob from the V_H_ domain. Interestingly, our in silico assessment did not find immunogenic peptides in NC-Cow1 Fab. No new immunogenic peptides were formed after grafting of the bovine CDRs or the bovine knob onto human scaffolds (Supplementary Fig. 13). Future work with animal models will have to further assess the immunogenicity of bovine antibodies with ultra-long CDRs and their humanized counterparts in vivo.

An interesting question related to the grafting of the bovine knob is how to define which residues from the ultra-long CDR-H3 have to be transplanted in order to maintain optimal antigen-binding properties. In our work, we used the knob and stalk definitions from an earlier publication^18^. The insertion points for the knob into human scaffolds are selected by inspecting the crystal structure of the human antibody with the aim to preserve residues that form the stalk in the wt human CDR-H3s. In future, the influence on the residues linking the knob and the stalk, as well as the influence on different insertion points, could be studied in more detail as these residues could impact the flexibility and orientation of the knob relative to the antibody framework. Likewise, the optimization of linkers that connect grafted mini-domains (e.g., from de novo design) to the CDR-H3 could be important.

The design principles revealed in the analysis of natural antibodies with ultra-long CDR-H3s inspired us to investigate other approaches for the discovery and grafting of complex CDRs. Such approaches could quickly identify knobs against antigens of concern. Ideally, the knobs will be isolated by in vitro display or in silico techniques like de novo design. We believe that knobs can be easily grafted on bovine and human antibody scaffolds that have a stalk with optimal length. We demonstrated the feasibility of such an approach by reformatting LCB1^34^, a de novo designed mini-domain against SARS-CoV-2 and used it as an ultra-long CDR-H3 in different Fabs (Fig. 6). The inserted mini-domain retained its high binding affinity for the RBD. Virus neutralization assays showed high neutralization efficiency not only for a SARS-CoV-2 strain that was isolated early in the pandemic, but also for the prevalent SARS-CoV-2 Alpha variant. A unique feature observed was the dimerization of LCB1 in the Fab context. As dimerization of Fabs improves their half-lives and reduces clearance^38^, our approach could allow the generation of antibody-based therapeutics with enhanced half-life but without Fc-effector functions. The strategy to insert de novo designed antigen-binding peptides as ultra-long CDRs will offer valuable alternatives to animal immunization and in vitro display techniques.

In conclusion, the elucidation of the design principles for maintaining ultra-long CDRs in antibodies revealed basic insight into antibody architecture and at the same time uncovered exciting opportunities for antibody design extending the repertoire in new directions.

## Methods

### Protein design and expression

The V_H_ and V_L_ domains of NC-Cow1^15^ were fused to bovine C_H_1 and C_L_ from other bovine Fab fragments with ultra-long CDR-H3^16^ to obtain the Fd and LC, respectively. The sequences for the Fd and LC of human Fabs are obtained from the Protein Data Bank (PDB) – trastuzumab (6B9Z), 32H+109L (5CEX), and PGT145 (3U1S). Mutants were designed as described in the results section. Signal peptides were fused on the N-terminal of each Fd (MDWTWRVFCLLAVAPGAHS) and LC (MAWSPLFLTLITHCAGSWA). The gene for the receptor-binding domain (RBD) of SARS-CoV-2 residues 319-541 (GenBank: QHD43416.1) with a 6xHis tag on the C-terminal. A Kozak sequence was added and the genes were optimized for *Homo sapiens* with the GeneArt tool and inserted in a pcDNA3.1(+) vector with HindIII and XhoI restriction sites. The vectors containing the genes were obtained by commercial gene synthesis and cloning from Thermo Fisher. The genes of the full-length IgGs were obtained by fusing the sequence for the respective Fd to the Fc of trastuzumab using overhang primers and sequence- and ligation-independent cloning^39^. The NEBuilder Assembly Tool (http://nebuilder.neb.com/#!/) was used to design the primers (Supplementary Table 1). The plasmids for the HIV-1 Env antigen (BG505 SOSIP.664 gp140-his) and furin were a kind gift from John P. Moore at Cornell University^40^. Larger amounts of the plasmids were purified with Midiprep kits (Promega) from overnight cultures with transformed XL1-Blue cells. The correct inserts in the purified plasmids were verified by sequencing.

All proteins were produced by transient expression in Expi293™ cells (Thermo Fisher) grown in Expi293™ expression medium at 37 °C with 8% CO_2_. The cells were transfected with 1 µg plasmid per 1 mL of cell suspension (2:1 LC/Fd plasmid ratio for the Fabs, 2:1 LC/HC ratio for full-length IgGs, 4:1 plasmid ratio for BG505 SOSIP.664/furin) using the ExpiFectamine™ 293 transfection kit or jetOPTIMUS® (Polyplus) following the manufacturers’ protocols. When jetOPTIMUS® was used, 1 mM sodium propionate and 5 mM sodium valproate were added to the cells 16–20 h after transfection in an analogical way to the addition of the enhancers from the ExpiFectamine™ 293 kit. The cell supernatants were collected by centrifugation and filtered six days after transfection.

The secreted Fabs were purified from the cell supernatants by affinity chromatography using CaptureSelect™ LC-lambda (ung) or IgG-CH1 affinity matrix (Thermo Fisher) for bovine and human Fabs respectively. For full-length human IgGs, we used protein A chromatography. The bound sample was washed with phosphate-buffer saline (PBS) and eluted with 0.1 M glycine pH 3.0. The samples were eluted into 1/5 volume of 1 M tris pH 8.5 at 2–8 °C. The His-tagged proteins were purified on a HisTrap HP column (Cytiva). The bound samples were washed with 20 mM sodium phosphate pH 7.4 with 500 mM NaCl and 20 mM imidazole and eluted with 20 mM sodium phosphate pH 7.4 with 500 mM NaCl and 500 mM imidazole. After affinity chromatography, the samples were further purified by size-exclusion chromatography with a HiLoad® Superdex 200 26/60 column (Cytiva) and PBS as running buffer. Finally, the proteins were concentrated with VivaSpins® (Sartorius), their concentration was determined by UV spectrometry and the samples were snap frozen for storage until used.

### Immunoprecipitation

The standard ExpiFectamine™ 293 protocol was used to transfect Expi293™ cells for immunoprecipitation. The transfections were performed in parallel on cells at the same passage to allow head-to-head comparisons. Six days post transfection, the cells were pelleted by centrifugation and 100 µL supernatant were mixed with 10 µL CaptureSelect™ LC-lambda (ung) slurry and incubated for 1 h at 37 °C at 1000 rpm in a ThermoMixer (Eppendorf). Subsequently, the beads were washed with three rounds of discarding the supernatant, resuspending the pellet in PBS and centrifugation. After washing, the pellet was resuspended in 40 µL 2x Lämli. The resuspended samples were denatured for 5 min at 95 °C, cooled to room temperature, centrifuged, and 10 µL supernatant was analyzed by sodium dodecyl sulfate-polyacrylamide gel electrophoresis on 4-20% gradient gels (Serva) using 50 mA current for 40 min. The gels were stained with colloidal Coomassie.

### Size-exclusion chromatography coupled to multi-angle light scattering (SEC MALS)

A Shimadzu HPLC equipped with a UV detector, refractive index detector and a HELEOS II MALS detector (Wyatt Technology) was used. The running buffer was PBS with a flow rate of 0.8 mL/min. The column was a Superdex 200 Increase 10/300 GL column (cytiva). The chromatograms were collected and evaluated with the Astra software v5 (Wyatt Technology).

### FUV CD spectroscopy

The far UV circular dichroism (FUV CD) spectra were collected at 20 °C with 1 mm quartz cuvettes at 0.1 mg/mL protein concentration. A Chirascan CD spectrometer (Applied Photophysics) was used to measure the spectra at 20 °C and thermal transitions of the NC-Cow1 mutants. A thermal ramp of 1 °C/min was used. Due to technical difficulties, a Jasco J-1500 spectropolarimeter was used to measure the FUV CD spectra if the human and chimeric Fabs. The protein concentration for FUV CD measurements was 0.1 mg/mL.

### Surface plasmon resonance (SPR)

A Biacore X-100 system (cytiva) was used to measure the binding affinities using HBS-EP + as the running buffer. The HIV-1 Env antigen was immobilized to around 1000 RU on CM5 chips. The corresponding analyte Fabs were injected in five different concentrations (1, 10, 25, 50 and 100 nM) over the immobilized HIV-1 Env antigen in multi-cycle kinetic mode. The ligand RBD from SARS-CoV-2 was immobilized to around 100 RU on CM5 chips. The BLC1-containing Fabs were injected in different concentrations (0.05, 0.5, 5, 12.5 and 50 nM) in multi-cycle kinetic mode. The sensorgrams were fit to a 1:1 binding model with the Biacore X-100 software.

### Differential scanning calorimetry (DSC)

All samples were measured with a Microcal PEAQ-DSC system (Malvern Panalytical). The volume of the solutions for the measurements was 250 µL for both the sample and reference cells. All measurements were performed with a temperature ramp of 1 °C/min to collect thermograms in the range from 20 °C to 100 °C. The thermograms were buffer subtracted.

### Fluorescence spectroscopy (nanoDSF)

The samples with a concentration of 0.1 mg/mL were filled in high sensitivity capillaries (NanoTemper Technologies) and sealed. The capillaries were placed in a Prometheus NT.48 (NanoTemper Technologies) and a temperature ramp of 1 °C/min was applied, while the intrinsic protein fluorescence intensity at 350 nm was measured after excitation at 280 nm. The PR. ThermControl V2.1 software was used to determine the apparent melting temperatures from the fluorescence intensity signal at 350 nm.

### Biolayer interferometry (BLI)

A Blitz (ForteBio) and HBS-EP + buffer were used for the BLI experiments. Human ACE2 receptor (residues 18-740) with an Fc tag and a concentration of 50 µg/mL was captured on Protein A BLI sensors for 300 s. Next, the sensor with captured ACE2 was placed into 1000 nM of SARS-CoV-2 RBD or 1000 nM SARS-CoV-2 RBD pre-mixed with 1000 nM Fab-LCB1 for an association phase of 300 s, followed by 300 s dissociation in HBS-EP + buffer. The Blitz software was used for data collection and analysis.

### Molecular dynamics (MD)

Molecular dynamics (MD) simulations were performed with the Amber18 simulation package^41^. The starting coordinates were taken from the crystal structure (PDB: 6OO0) and included only the variable domains of the Fab fragment (residues 1-107 of chain L and 1-165 of chain H) with the knob structure. Mutations of the Cys residues in the knob region (to Ala) to eliminate disulfide bridges and substitution of the stalk to Gly (G-stalk variant) were generated in silico using the leap module of Amber18. Each protein was solvated in TIP3P water^42^ in a periodic octahedron box with a minimum distance of protein atoms to the box boundary of 10 Å. The ff14SB force field^43^ was employed and Na^+^ and Cl^−^ ions were added to neutralize the system and reach an ion concentration of 0.15 M. Energy minimization of each system was performed with the sander module of Amber18 (1500 minimization cycles). The systems were heated in steps of 100 K (20 ps per step) to a final temperature of 300 K with the solute non-hydrogen atoms harmonically restraint to the start structure. All bonds involving hydrogen atoms were kept at optimal length. In additional 4 steps the harmonic restraints were removed stepwise. For the production simulations (i.e. started from structures at equilibrium gained from the previous equilibration phase) hydrogen mass repartitioning (HMR)^44^ was employed allowing a time step of 4 fs (instead of 2 fs used during heating and equilibration). Unrestrained production simulations were extended to 0.5 µs for each system. Coordinates were saved every 8 ps. Analysis of trajectories was performed using the Amber cpptraj module.

### Virus strains

SARS-CoV-2 (EPI_ISL_582134) and SARS-CoV-2 B.1.1.7 (EPI_ISL_755639) were isolated from patient material in Germany. Briefly, SARS-CoV-2 was isolated from a COVID-19 infected patient who was among the first documented cases in Germany at the end of January 2020 (Webasto cluster outbreak). This strain already contains the S1 D614G mutation. SARS-CoV-2 and SARS-CoV-2 B.1.1.7 were isolated and passaged in Vero E6 cells (derived from African green monkey kidney epithelial cells). DMEM medium (10% fetal calf serum (FCS), 1% penicillin/streptomycin (P/S), 200 mmol/L L-glutamine, 1% MEM-Non-Essential Amino Acids (NEAA), 1% sodium pyruvate (all from Gibco) was used as culture media. Viral titer was determined by Plaque Assay^45^.

### Viral neutralization assay

Vero E6 cells (ATCC-CRL-1586) were seeded at 1.6*10^4^ cells/well in a 96-well plate in DMEM medium supplemented with 10% FCS, 1% P/S, 200 mmol/L L-glutamine, 1% NEAA, 1% sodium pyruvate (all from Gibco) and incubated overnight at 37 °C, 5% CO_2_. Serial dilutions of constructs were prepared in fresh media and pre-incubated with the virus for 1 h at 37 °C. Vero E6 cells were infected with the viral neutralization mix with a multiplicity of infection (MOI) of 0.03. After 1 h incubation at 37 °C, inoculum was replaced with fresh medium and the plates were incubated for 24 h at 37 °C. Uninfected Vero E6 cells which were incubated with culture media represents mock cells. On the next day, supernatant was discarded and the cells were washed with PBS. The cells were fixed with 4% paraformaldehyde (ChemCruz) for 15 min at room temperature (RT). Following a washing step, cells were permeabilized with PBS buffer supplemented with 0.5% saponin (Roth) for 15 min at RT. Subsequent in-cell ELISA was started with the blocking of the cells with 0.1% saponin-PBS buffer containing 10% goat serum (Sigma) overnight at 4 °C. On the next day, Vero E6 cells were incubated with a 1:1500 dilution of anti-SARS-CoV-2-N T62 antibody (SinoBiological 40143-T62) with 1% FCS-PBS for 2 h at RT. Following a washing step with PBS supplemented with 0.05% Tween-20 (Roth), cells were incubated with a 1:4000 dilution of goat anti-rabbit IgG2a-HRP (EMD Millipore 12-348) with 1% FCS-PBS for 1 h at RT. After washing the plates, 3,3′,5,5′-tetramethylbenzidin substrate (Invitrogen) was added and incubated for 15-20 min at RT. The color reaction was stopped by adding 2 N H_2_SO_4_ (Roth). Colorimetric detection was performed on a Tecan infinite F200 pro plate reader (Tecan) at 450 nm and at 560 nm.

The neutralization data was fitted with GraphPad Prism 9 to obtain the IC50 values from the following equation (1):

1 $${{{{{\rm{Y}}}}}}={{{{{\rm{Bottom}}}}}}+({{{{{\rm{Top}}}}}}-{{{{{\rm{Bottom}}}}}})/(1+({{{{{\rm{IC}}}}}}50/{{{{{\rm{X}}}}}}){}^{\wedge }{{{{{\rm{HillSlope}}}}}})$$

where X is the protein concentration, Y is the neutralization efficiency in %, top and bottom are the plateaus in the same units as Y, HillSlope is a slope factor, IC50 is the 50% inhibitory concentration.

### Data analysis

Origin Lab (v2019) was used for the calculation of mean values and standard deviations.

### Reporting summary

Further information on research design is available in the Nature Research Reporting Summary linked to this article.

## Supplementary information

Supplementary Information

Reporting summary

## Data Availability

Source data are provided with this paper. Other data that support the findings of this study are available from the corresponding authors upon reasonable request. Source data are provided with this paper.

## Source data

Source Data
