# Supplementary material for: Mechanistic principles of an ultra-long bovine CDR reveal strategies for antibody design
Source: Nat Commun. 2021 Nov 18;12:6737. doi: 10.1038/s41467-021-27103-z (PMC8602281; doi:10.1038/s41467-021-27103-z)
Supplement: Supplementary file 1 — Supplementary Information [file 41467_2021_27103_MOESM1_ESM.pdf]

Supplementary Information

Mechanistic principles of an ultra-long bovine CDR reveal strategies for antibody design

Hristo L. Svilenov[1*], Julia Sacherl[2], Ulrike Protzer[2,3], Martin Zacharias[4], Johannes Buchner[1,*]

[1]Center for Protein Assemblies and Department Chemie, Technische Universität München, 85748 Garching, Germany

[2] Institute of Virology, Technical University of Munich / Helmholtz Zentrum Munich, Munich, Germany

[3] German Center for Infection Research, Munich partner site, Munich, Germany

[4]Center for Protein Assemblies and the Department Physik, Technische Universität München, 85748 Garching, Germany

* To whom correspondence should be addressed:

hristo.svilenov@tum.de

johannes.buchner@tum.de

Supplementary Figure 1

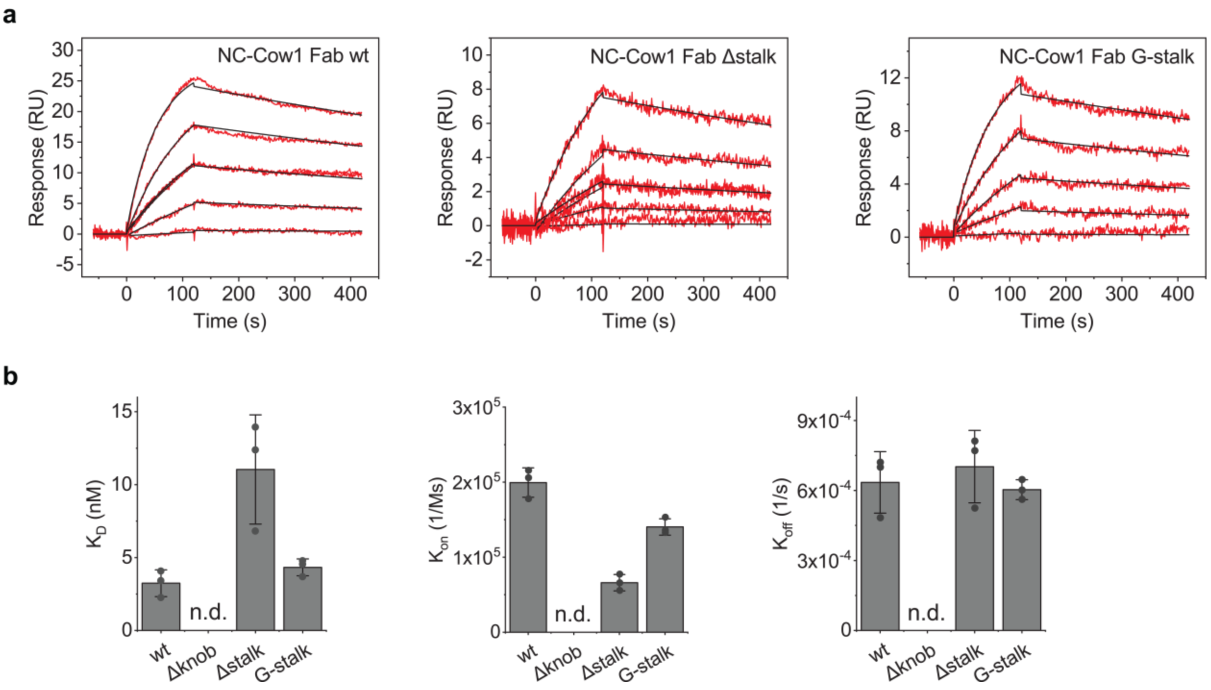

Supplementary Figure 1. Binding of NC-Cow1 wt and mutants to the HIV-1 Env protein. a Exemplary multi-cycle kinetic SPR data (red traces) with fits (black lines) to a 1:1 binding model. **b** Kinetic constants from the data in **a**. The bars are mean of triplicates with standard deviations. The circles are individual measurements.

Supplementary Figure 2

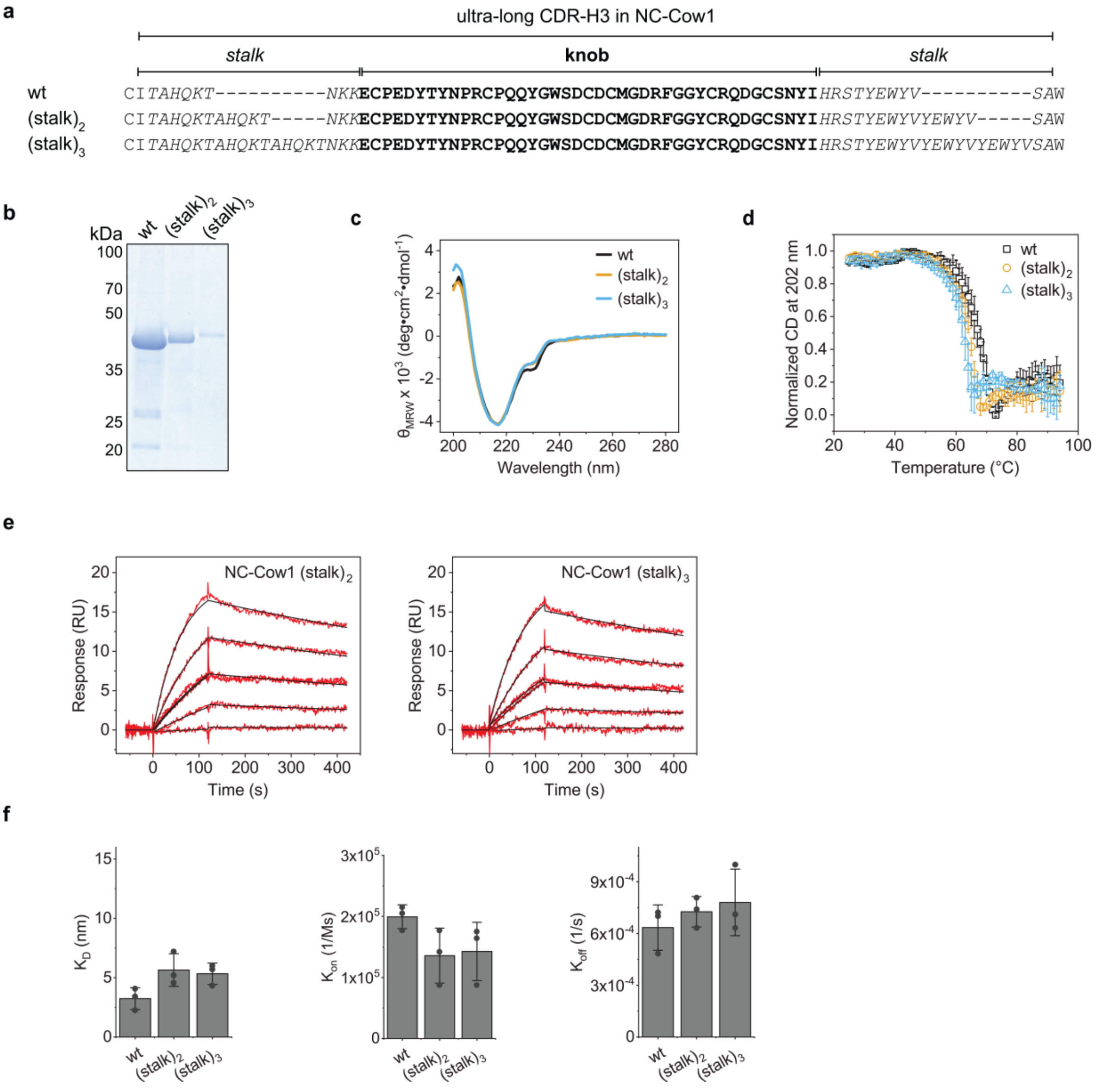

Supplementary Figure 2. Stalk extensions in the ultra-long CDR-H3 of NC-Cow1 Fab. a The $(stalk)_2$ and $(stalk)_3$ mutants are designed by repeating complementary ascending and descending strands of the stalk. **b** Immunoprecipitation of Expi293 supernatants after transient expression of Fab fragments followed by SDS-PAGE**.** Two independent experiments. **c** FUV CD spectra and **d** thermal stability of the NC-Cow1 Fab variants with extended stalk. Mean of triplicates with standard deviations. **e** Binding of the variants to the HIV-1 Env protein. Exemplary multi-cycle kinetic SPR data (red traces) with fits (black lines) to a 1:1 binding model. **f** Kinetic constants from the data in **e**. The bars are mean of triplicates with standard deviations. The circles are individual measurements.

Supplementary Figure 3

a

CDR-H3 replacement in NC-Cow1

wt→BS1	CAKSSGTNFAVATWDVIDAW
wt→BS2	CAKSSGNVGFYQSYNSRSWKQYVDAW
wt→B4	CAKCSVGDSGSYACTGRKGEYVDAW
wt→B13	CATLGCYFVEGVGYDCTYGLQHTTFHDAW

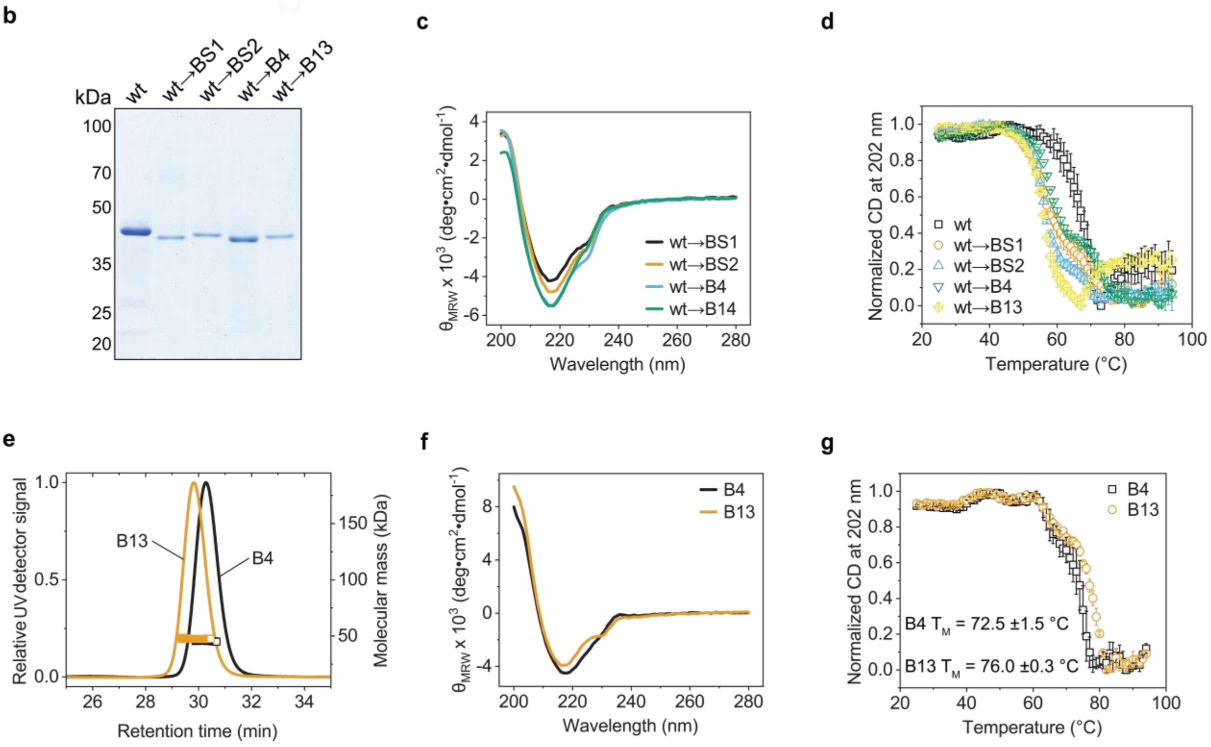

Supplementary Figure 3. Properties of NC-Cow1 Fabs with grafted short bovine CDR-H3s and two corresponding bovine Fabs with a short CDR-H3 in wildtype. a Sequences of the short bovine CDR-H3s that were used to replace the ultra-long CDR-H3**.** The cysteine residues in CDR-H3 are underlined. **b** Immunoprecipitation of Expi293 supernatants after transient expression of Fab fragments followed by SDS-PAGE. Two independent experiments. **c** FUV CD spectra and **d** thermal stability of the NC-Cow1 Fab mutants with short bovine CDR-H3s. Mean of triplicates with standard deviations. **e** Molecular mass and eluting peaks in SEC-MALS (0.5 mL/min flow rate), **f** FUV CD spectra and **g** thermal stability of bovine Fabs with short CDR-H3s in wildtype. Mean of triplicates with standard deviations. The melting temperatures are mean values of triplicates with standard deviations.

Supplementary Figure 4

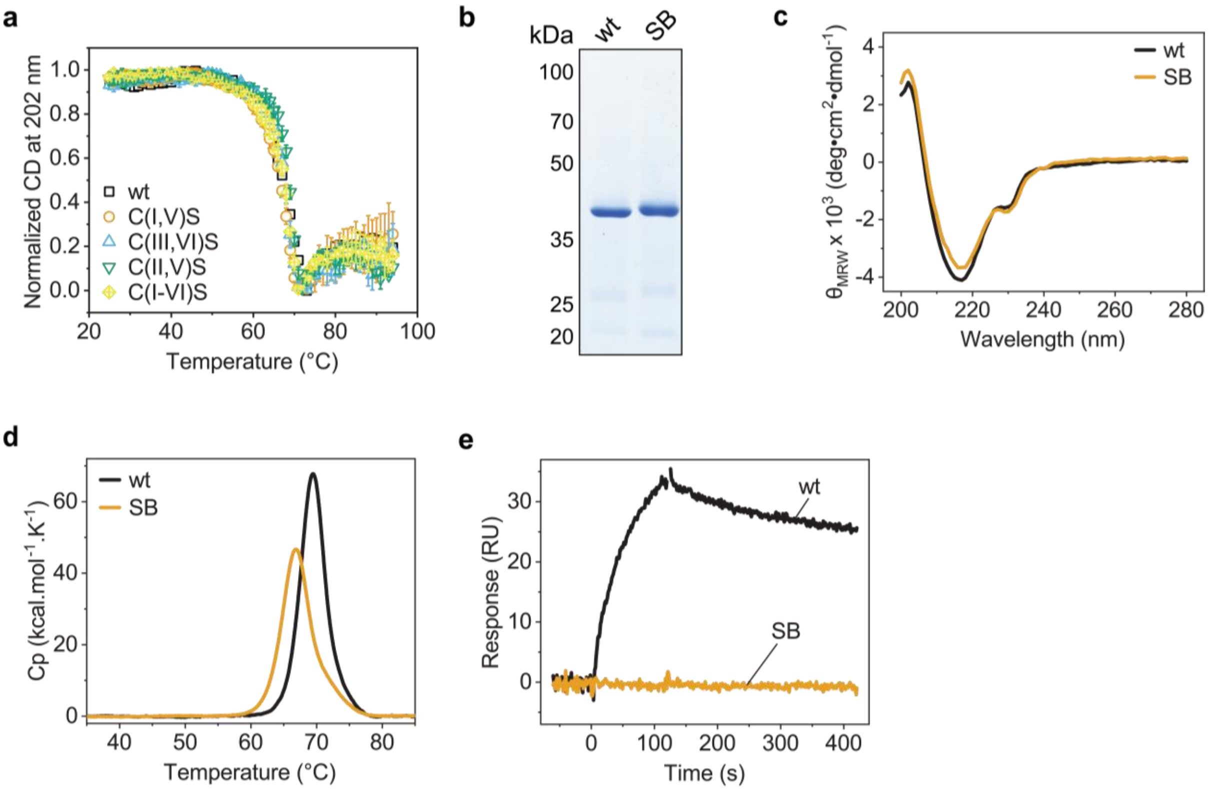

Supplementary Figure 4. Properties of NC-Cow1 variants with replaced disulfide bonds in the knob. a Thermal unfolding of C→S knob mutants tracked by FUV CD**.** Mean values of triplicates with standard deviation. **b** Immunoprecipitation of Expi293 supernatants after transient expression of Fab fragments (NC-Cow1 wt or NC-Cow1 with one disulfide bond replaced by a salt bridge (SB)) followed by SDS-PAGE**.** Two independent experiments. **c** FUV CD spectra and **d** thermal stability by DSC of NC-Cow1 SB compared to wt. **e** The SB mutant (100 nM) shows no binding to the HIV-1 Env antigen in SPR.

Supplementary Figure 5

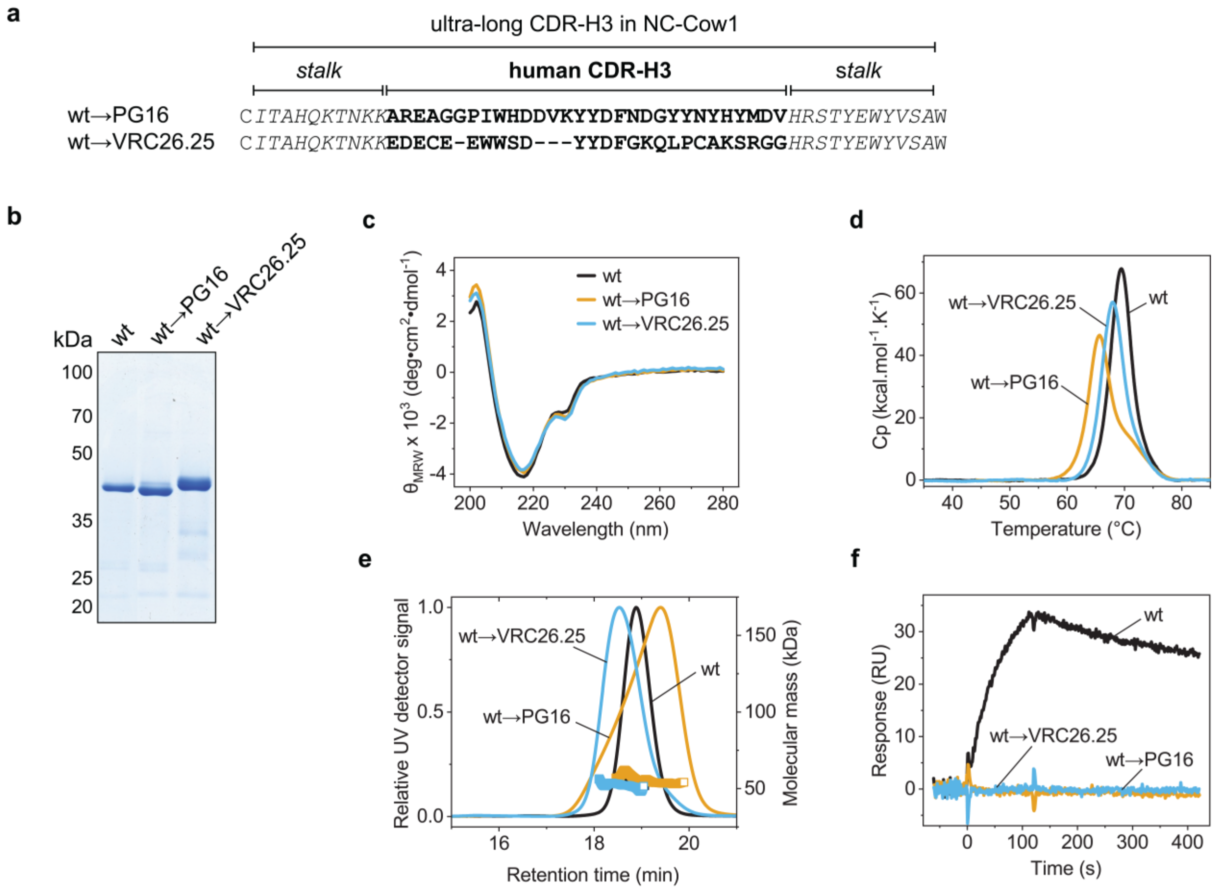

Supplementary Figure 5. Properties of NC-Cow1 Fabs where the bovine knob is replaced by loops from long human CDR-H3s. **a** Human sequences from the CDR-H3 of PG16 and VRC26.25 used to replace the knob in NC-Cow1. **b** Immunoprecipitation of Expi293 supernatants after transient expression of Fab fragments followed by SDS-PAGE. Two independent experiments. **c** FUV CD spectra and **d** thermal stability of the NC-Cow1 Fab mutants. **e** Molecular mass and eluting peaks in SEC-MALS. **f** Binding of 100 nM from the Fabs to the HIV Env protein in SPR.

Supplementary Figure 6

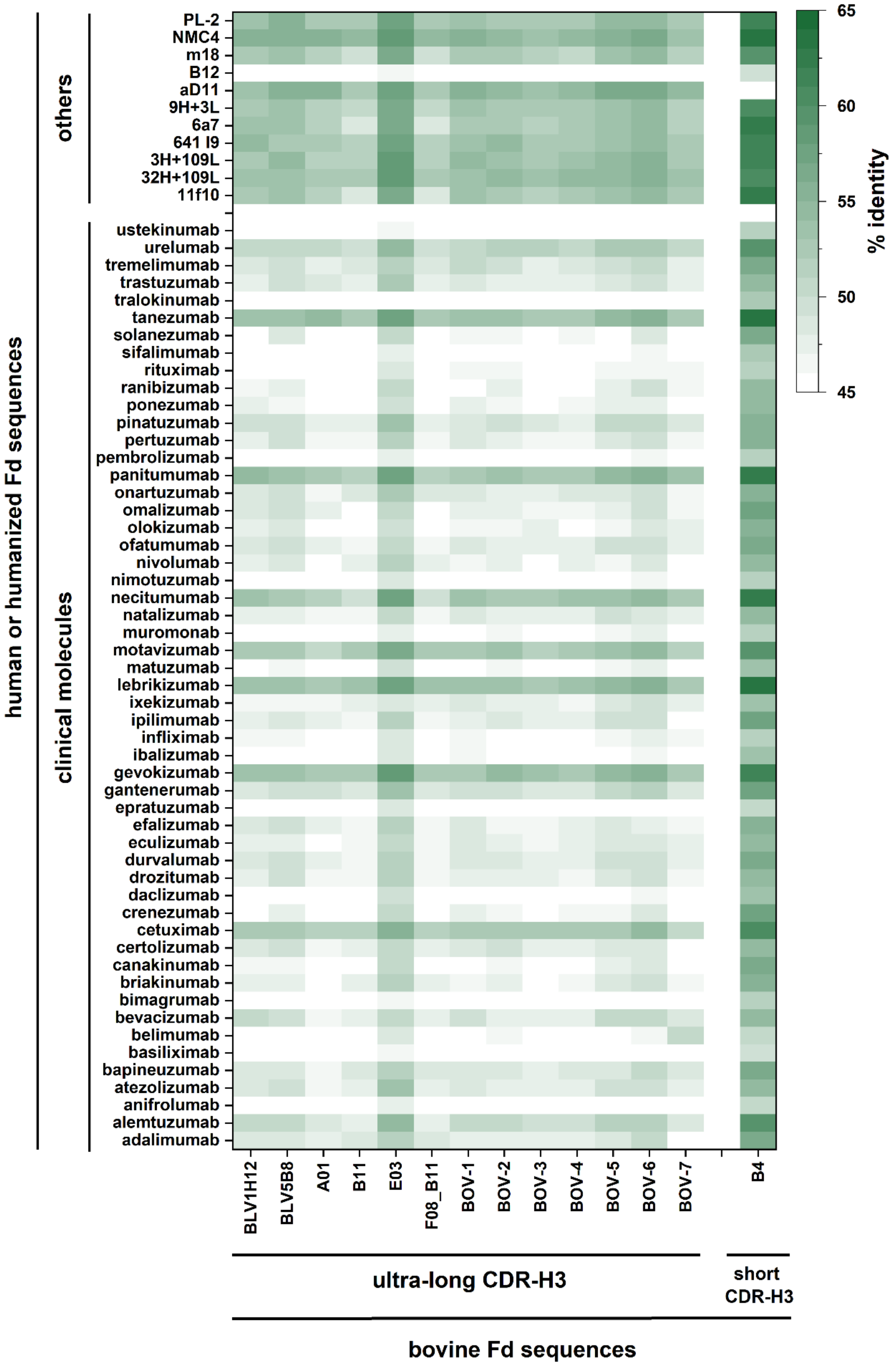

Supplementary Figure 6. Protein BLAST sequence percent identity between the Fd chains of human antibodies and bovine antibodies

Supplementary Figure 7

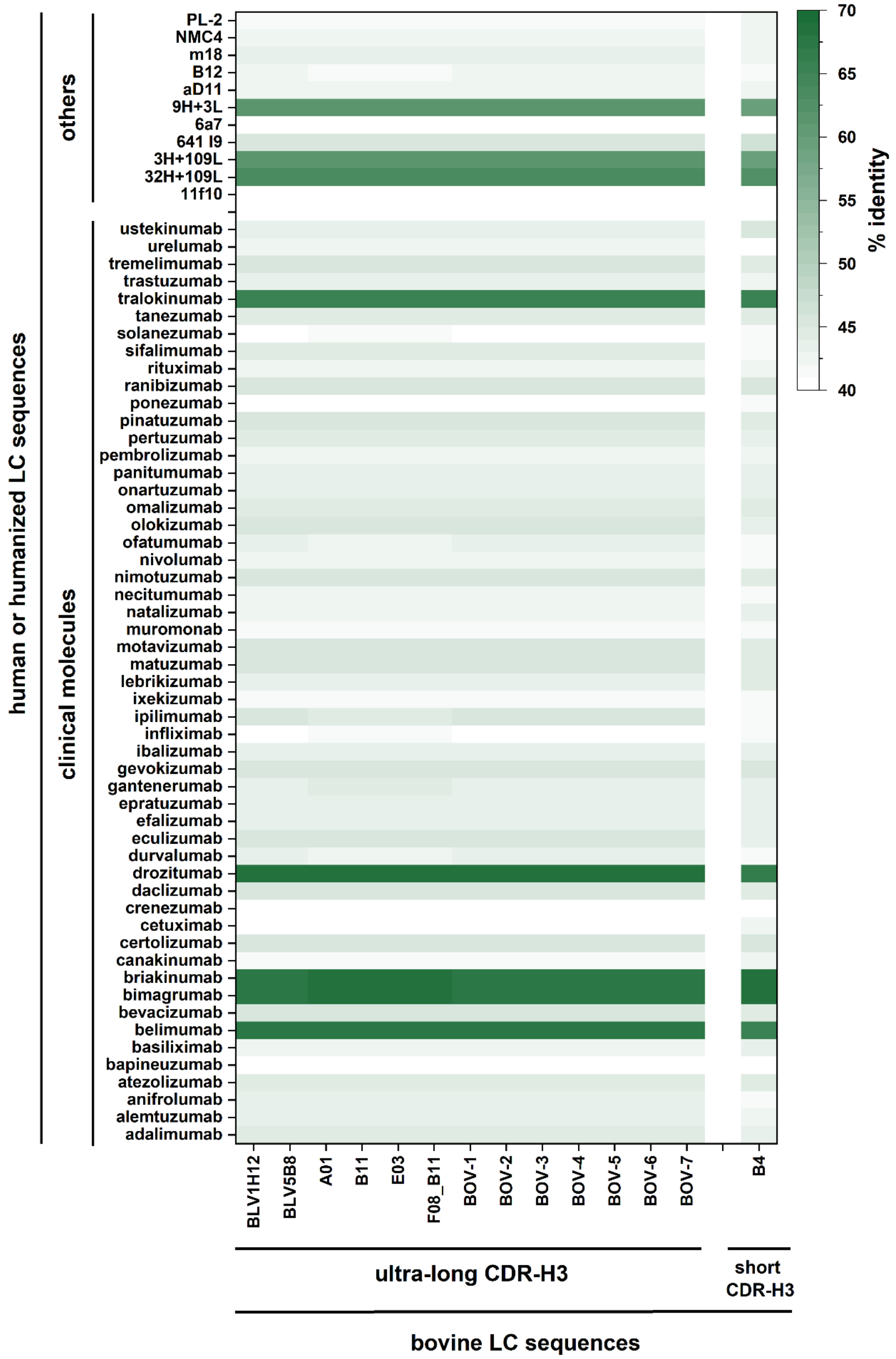

Supplementary Figure 7. Protein BLAST sequence percent identity between the LCs of human antibodies and bovine antibodies

Supplementary Figure 8

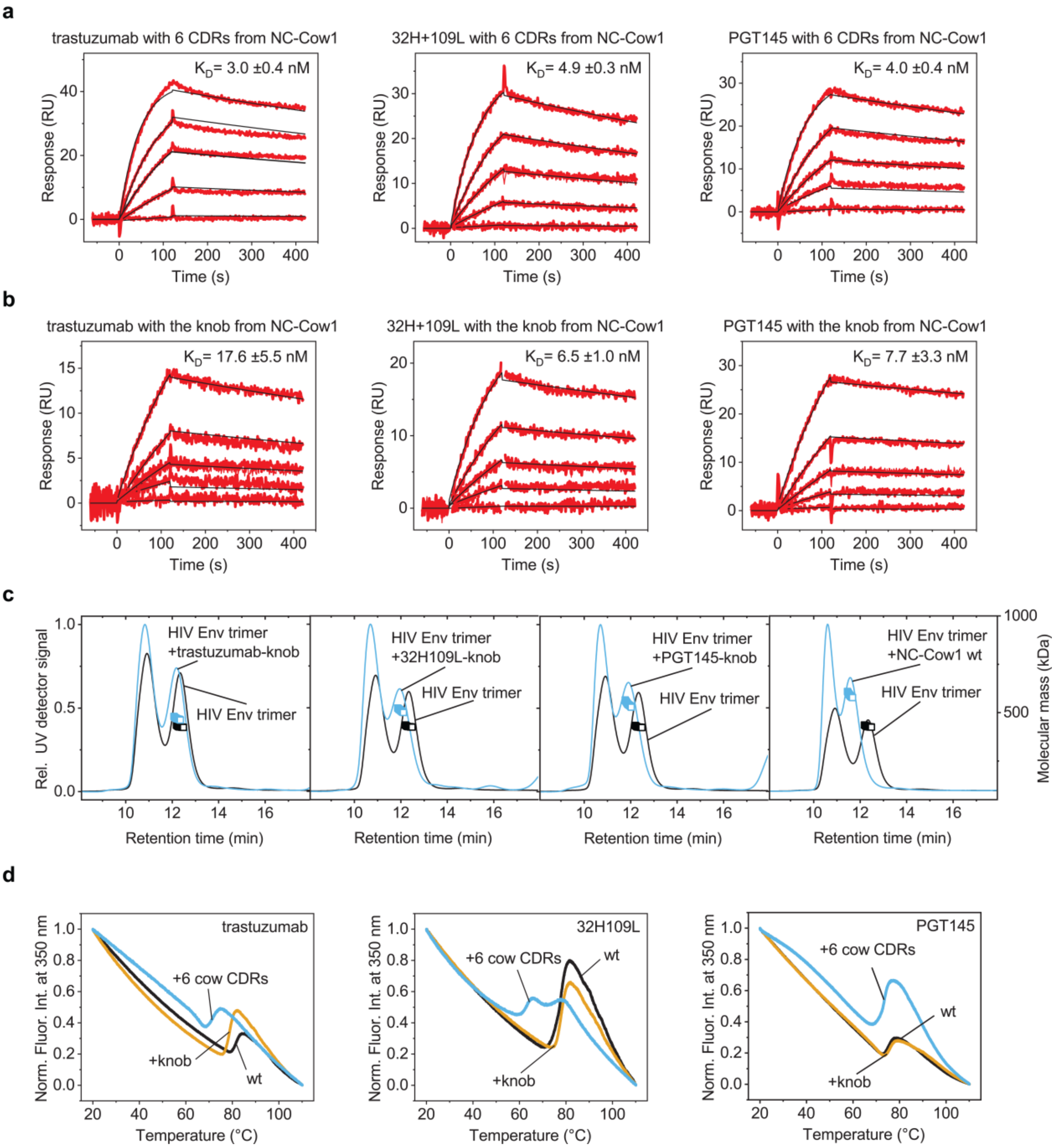

Supplementary Figure 8. Properties of chimeric Fab fragments obtained by grafting the 6 CDRs or the knob from NC-Cow1 to human scaffolds. a and **b** Exemplary multi-cycle kinetic SPR data (red traces) with fits (black lines) to a 1:1 binding model with K_D values. Mean of triplicates with standard deviation. **c** Binding of the chimeric constructs (human Fab with bovine knob from NC-Cow1) to a soluble HIV Env trimer detected by SEC-MALS. **d** Thermal stability of wt and chimeric constructs studied by the change in the intrinsic protein fluorescence during heating.

Supplementary Figure 9

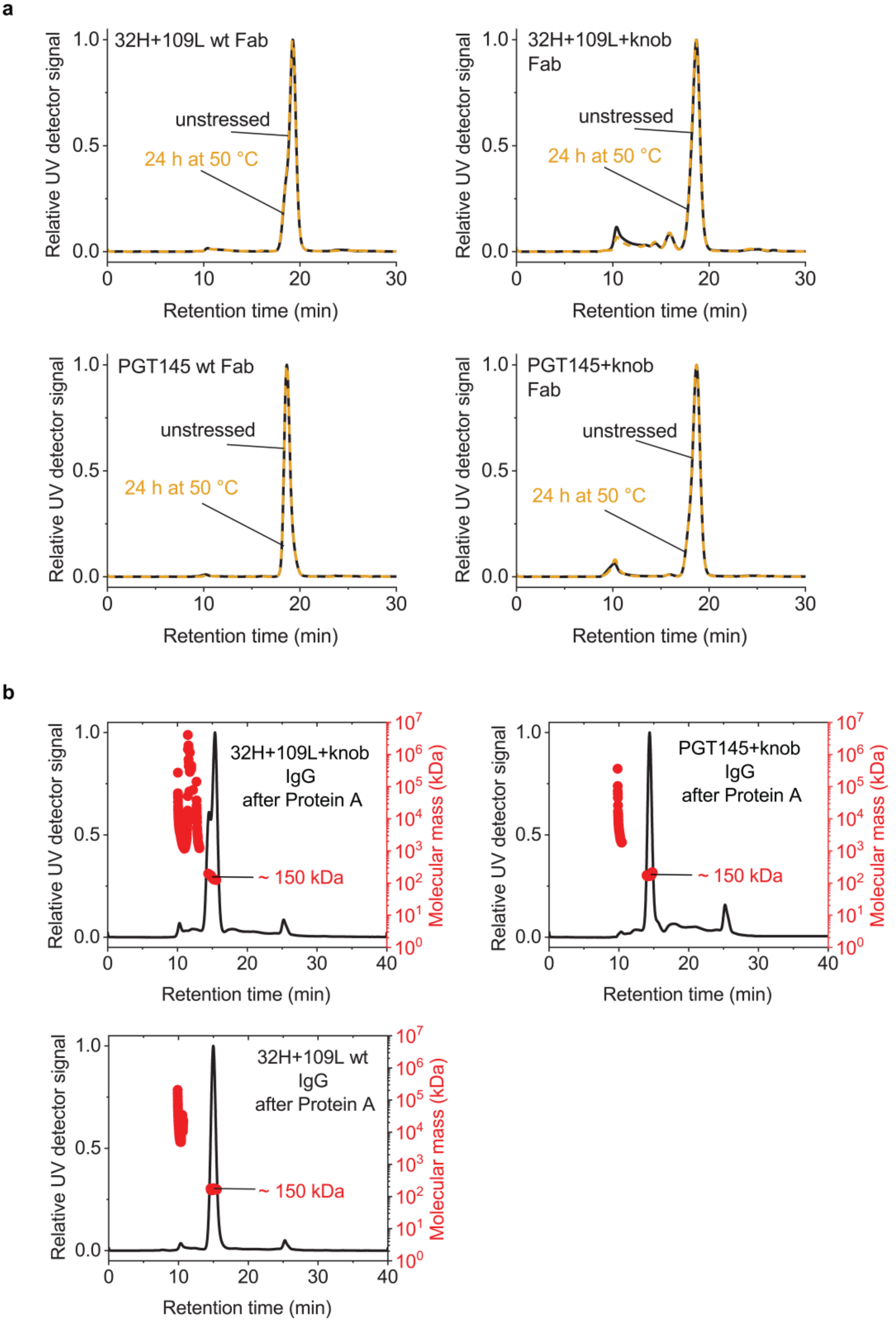

Supplementary Figure 9. Aggregate analysis of chimeric and wt constructs. a SEC analysis of the purified wt and knob mutants. Unstressed (black line) or after incubation for 24 hours at 50 °C (yellow dashed line). **b** SEC-MALS analysis of full-length IgG constructs that were purified only with protein A chromatography. The main peak in the chromatograms has the molecular mass expected for an IgG.

Supplementary Figure 10

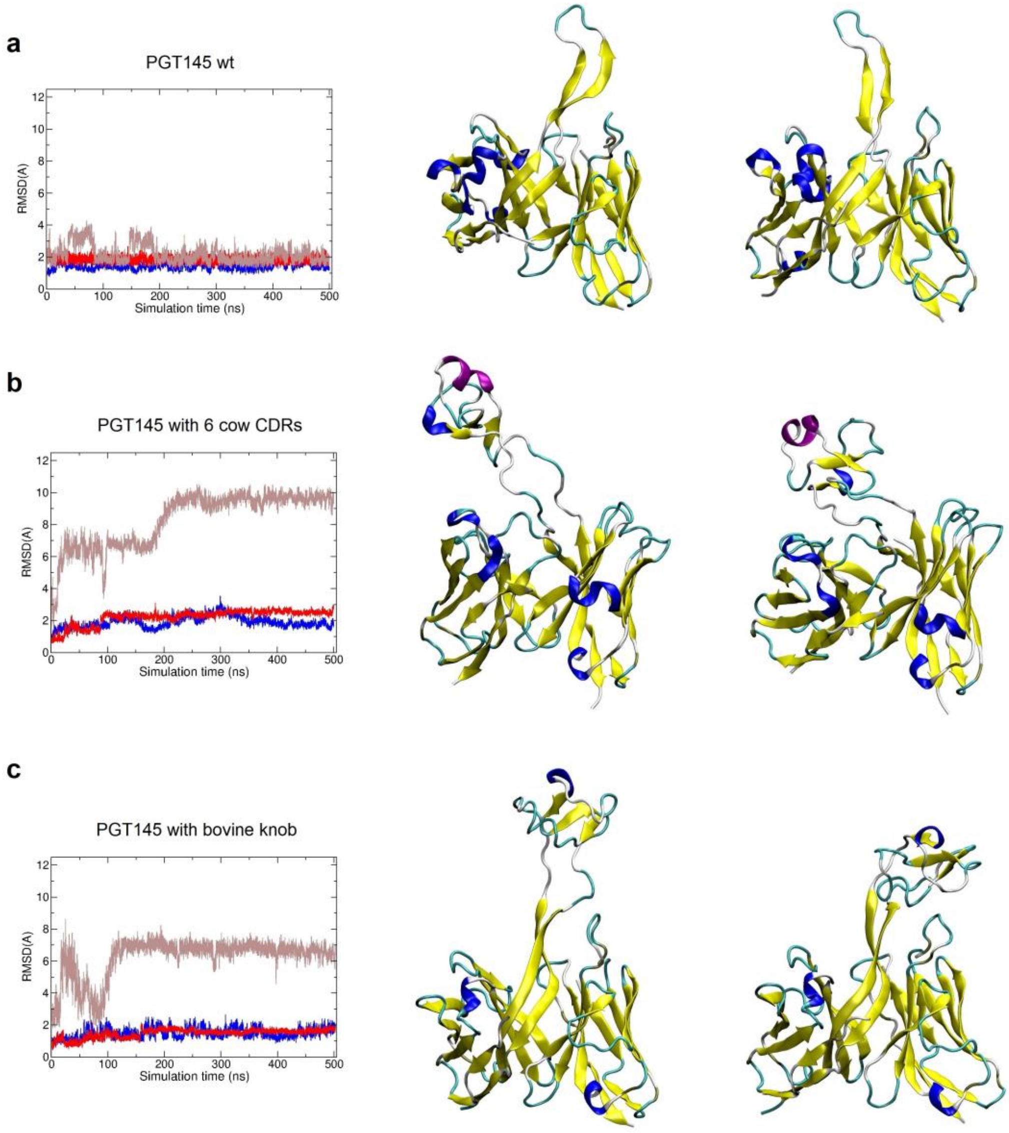

Supplementary Figure 10. Comparative MD-simulation on human and chimeric constructs. a Fv of PGT145 wt. **b** Fv of PGT145 with the 6 CDRs from NC-Cow1. **c** PGT145 with the bovine knob grafted onto the CDR-H3. The coloring of the RMSD is: Fv framework alone (blue), the knob alone (red), or the motion of the whole structure (brown). In the case of PGT145 wt, the red curve is for the beta hairpin in CDR-H3. The structures are snapshots from the respective simulation.

Supplementary Figure 11

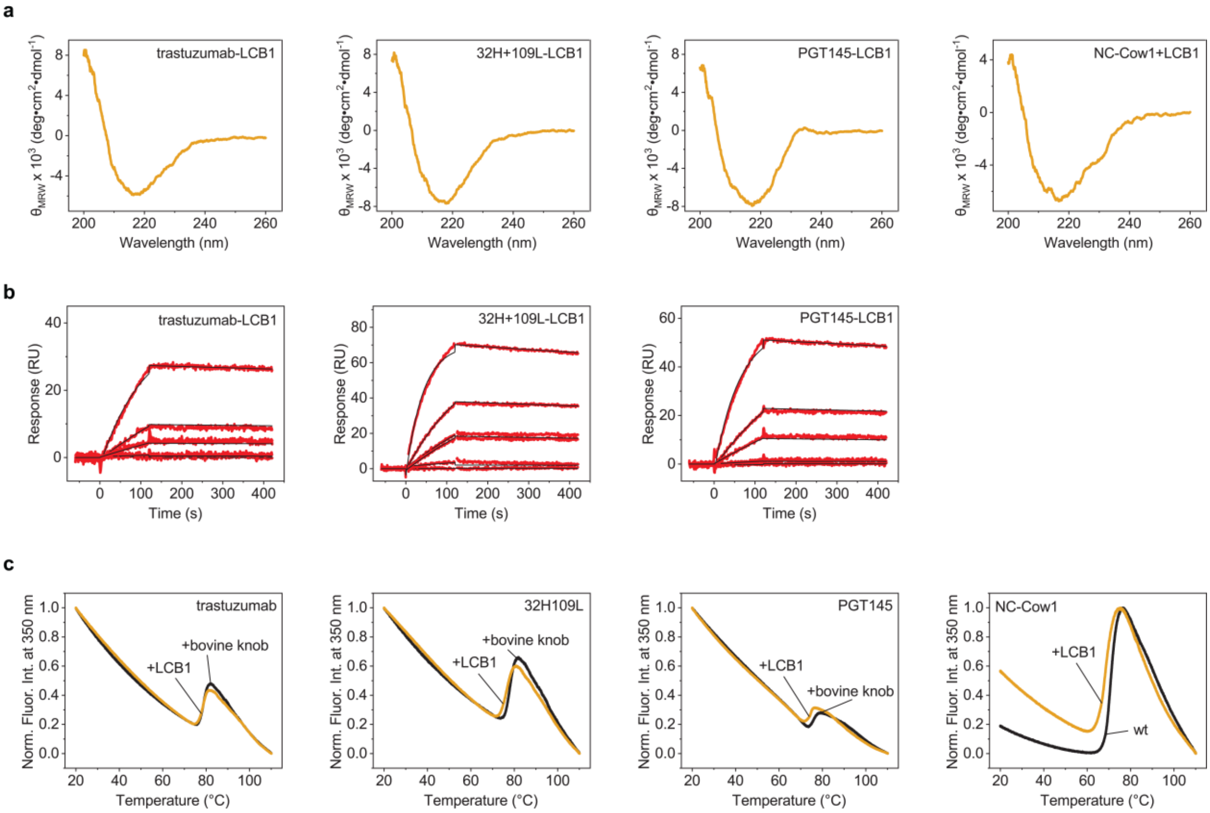

Supplementary Figure 11. Properties of Fab fragments containing a mini-domain from LCB1 in their CDR-H3s. a FUV CD spectra of the four proteins. **b** Exemplary multi-cycle kinetic SPR data (red traces) with fits (black lines) to a 1:1 binding model to characterize the binding between the Fab-LCB1s and the SARS-CoV-2 RBD. **c** Thermal stability of the Fab-LCB1s and the corresponding Fab with a bovine knob insertion in CDR-H3.

Supplementary Figure 12

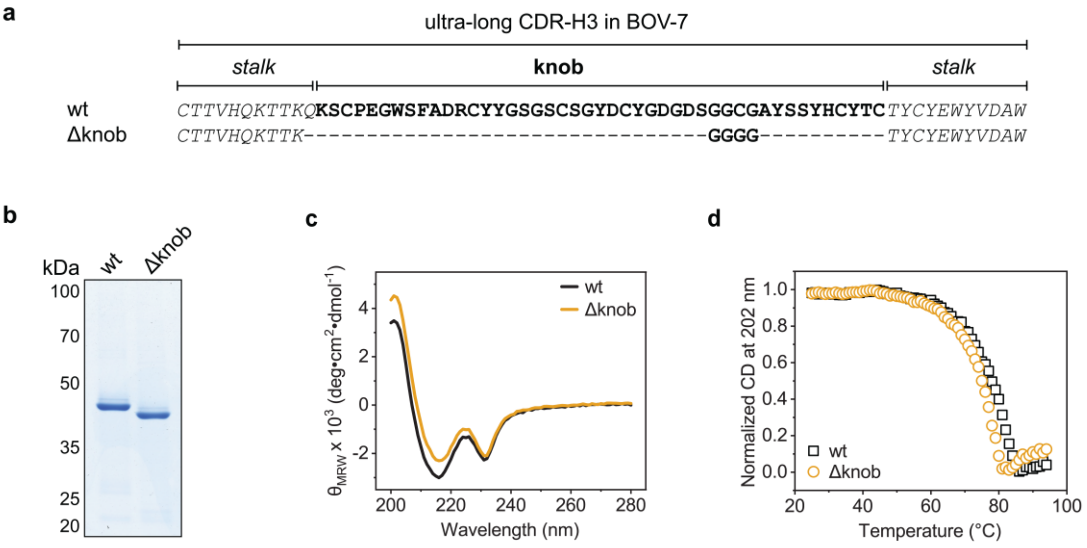

Supplementary Figure 12. Features of BOV-7 Fab and a mutant with knob deletion (Δknob). a Sequence alignment of the CDR-H3 in BOV-7 wt and BOV-7 Δknob. **b** Immunoprecipitation of Expi293 supernatants after transient expression of the Fab fragments followed by SDS-PAGE. Two independent experiments. **c** FUV CD spectra and **d** thermal stability of the BOV-7 wt and Δknob. Data are mean of duplicates.

Supplementary Figure 13

CLUSTAL O(1.2.4) multiple sequence alignment

Underlined residues indicate immunogenic sequences assessed by
http://tools.iedb.org/deimmunization/

```
                                                    CDR-H1                   CDR-H2
NC-Cow1_Fd               QVQLRESGPSLMKPSQTLSLTCTVSGSSLNDKSVGWVRQAPGKALQWLGSVDT-SGNTDY 59
trastuzumab_Fd           EVQLVESGGGLVQPGGSLRLSCAASGFNIKDTYIHWVRQAPGKGLEWVARIYPTNGYTRY 60
trastuzumab_Fd_knob      EVQLVESGGGLVQPGGSLRLSCAASGFNIKDTYIHWVRQAPGKGLEWVARIYPTNGYTRY 60
trastuzumab_Fd_cowCDRs   EVQLVESGGGLVQPGGSLRLSCAASGSSLNDKSVGWVRQAPGKGLEWVASVDT-SGNTDY 59
32H109L_Fd               QVQLQESGPGLVKPSETLSLTCSVSGASISDHYWSWIRQSPGKGLEWIGYVYD-SGDTNY 59
32H109L_knob_Fd          QVQLQESGPGLVKPSETLSLTCSVSGASISDHYWSWIRQSPGKGLEWIGYVYD-SGDTNY 59
32H109L_Fd_cowCDRs       QVQLQESGPGLVKPSETLSLTCSVSGSSLNDKSVGWIRQSPGKGLEWIGSVDT-SGNTDY 59
PGT145_Fd                QVQLVQSGAEVKKPGSSVKVSCKASGNSFSNHDVHWVRQATGQGLEWMGWMSHEGDKTGL 60
PGT145_knob_Fd           QVQLVQSGAEVKKPGSSVKVSCKASGNSFSNHDVHWVRQATGQGLEWMGWMSHEGDKTGL 60
PGT145_Fd_cowCDRs        QVQLVQSGAEVKKPGSSVKVSCKASGSSLNDKSVGWVRQATGQGLEWMGSVDT-SGNTDY 59
                         :*** :**  : :*. :: ::* .** .:.:    *:**: *:.*:*:. :   .. *


NC-Cow1_Fd               NPGLKSRLSITKDNSKSRISLTVTGMTTEDSATYYCITAH---QKTNKKECPEDYTYNPR 116
trastuzumab_Fd           ADSVKGRFTISADTSKNTAYLQMNSLRAEDTAVYYCSRWG-------GD----------- 102
trastuzumab_Fd_knob      ADSVKGRFTISADTSKNTAYLQMNSLRAEDTAVYYCSRWG-------GDECPEDYTYNPR 113
trastuzumab_Fd_cowCDRs   NPGLKSRFTISADTSKNTAYLQMNSLRAEDTAVYYCSRAH---QKTNKKECPEDYTYNPR 116
32H109L_Fd               NPSLKSRVNLSLDTSKNQVSLSLTAVTAADSAIYYCARTQH--GRRIYGIV--------- 108
32H109L_knob_Fd          NPSLKSRVNLSLDTSKNQVSLSLTAVTAADSAIYYCARTQH--GRRIYGECPEDYTYNPR 117
32H109L_Fd_cowCDRs       NPGLKSRVNLSLDTSKNQVSLSLTAVTAADSAIYYCARAH---QKTNKKECPEDYTYNPR 116
PGT145_Fd                AQKFQGRVTITRDSGASTVYMELRGLTADDTAIYYCLTGSKHRLRDYFLYNEY------- 113
PGT145_knob_Fd           AQKFQGRVTITRDSGASTVYMELRGLTADDTAIYYCLTGSKHRLRDYFLECPEDYTYNPR 120
PGT145_Fd_cowCDRs        NPGLKSRVTITRDSGASTVYMELRGLTADDTAIYYCLTAH---QKTNKKECPEDYTYNPR 116
                            .:.*..:: *.. .   :  : .: : *:* ***


                               CDR-H3
NC-Cow1_Fd               CPQQYGWSDCDCMGDRFGGYCRQDGCSNYI--HRSTYEWYVSAWGQGLLVTVSSASTTAP 174
trastuzumab_Fd           --------------------------------GFY----AMDYWGQGTLVTVSSASTKGP 126
trastuzumab_Fd_knob      CPQQYGWSDCDCMGDRFGGYCRQDGCSNYI--GFY----AMDYWGQGTLVTVSSASTKGP 167
trastuzumab_Fd_cowCDRs   CPQQYGWSDCDCMGDRFGGYCRQDGCSNYI--HRSTYEWYVSAWGQGTLVTVSSASTKGP 174
32H109L_Fd               ---------------------------AFR--EWFTY-FYMDVWGQGTPVTVSSASTKGP 138
32H109L_knob_Fd          CPQQYGWSDCDCMGDRFGGYCRQDGCSNYI--EWFTY-FYMDVWGQGTPVTVSSASTKGP 174
32H109L_Fd_cowCDRs       CPQQYGWSDCDCMGDRFGGYCRQDGCSNYI--HRSTYEWYVSAWGQGTPVTVSSASTKGP 174
PGT145_Fd                ---------------------------GPNYEEWGDYLATLDVWGHGTAVTVSSASTKGP 146
PGT145_knob_Fd           CPQQYGWSDCDCMGDRFGGYCRQDGCSNYIYEEWGDYLATLDVWGHGTAVTVSSASTKGP 180
PGT145_Fd_cowCDRs        CPQQYGWSDCDCMGDRFGGYCRQDGCSNYI--HRSTYEWYVSAWGHGTAVTVSSASTKGP 174
                                                                 :. **:*  ********..*


NC-Cow1_Fd               KVYPLSSCCGDKSSSTVTLGCLVSSYMPEPVTVTWNSGALKSGVHTFPAVLQSSGLYSLS 234
trastuzumab_Fd           SVFPLAPSSKSTSGGTAALGCLVKDYFPEPVTVSWNSGALTSGVHTFPAVLQSSGLYSLS 186
trastuzumab_Fd_knob      SVFPLAPSSKSTSGGTAALGCLVKDYFPEPVTVSWNSGALTSGVHTFPAVLQSSGLYSLS 227
trastuzumab_Fd_cowCDRs   SVFPLAPSSKSTSGGTAALGCLVKDYFPEPVTVSWNSGALTSGVHTFPAVLQSSGLYSLS 234
32H109L_Fd               SVFPLAPSSKSTSGGTAALGCLVKDYFPEPVTVSWNSGALTSGVHTFPAVLQSSGLYSLS 198
32H109L_knob_Fd          SVFPLAPSSKSTSGGTAALGCLVKDYFPEPVTVSWNSGALTSGVHTFPAVLQSSGLYSLS 234
32H109L_Fd_cowCDRs       SVFPLAPSSKSTSGGTAALGCLVKDYFPEPVTVSWNSGALTSGVHTFPAVLQSSGLYSLS 234
PGT145_Fd                SVFPLAPSSKSTSGGTAALGCLVKDYFPEPVTVSWNSGALTSGVHTFPAVLQSSGLYSLS 206
PGT145_knob_Fd           SVFPLAPSSKSTSGGTAALGCLVKDYFPEPVTVSWNSGALTSGVHTFPAVLQSSGLYSLS 240
PGT145_Fd_cowCDRs        SVFPLAPSSKSTSGGTAALGCLVKDYFPEPVTVSWNSGALTSGVHTFPAVLQSSGLYSLS 234
                         .*:**: .. ..*..*.:*****..*:******:******.*******************


NC-Cow1_Fd               SMVTVPGSTSG-QTFTCNVAHPASSTKVDKAVEPKSCDGS  273
trastuzumab_Fd           SVVTVPSSSLGTQTYICNVNHKPSNTKVDKKVEPKSCD--  224
trastuzumab_Fd_knob      SVVTVPSSSLGTQTYICNVNHKPSNTKVDKKVEPKSCD--  265
trastuzumab_Fd_cowCDRs   SVVTVPSSSLGTQTYICNVNHKPSNTKVDKKVEPKSCD--  272
32H109L_Fd               SVVTVPSSSLGTQTYICNVNHKPSNTKVDKKVEPKSCD--  236
32H109L_knob_Fd          SVVTVPSSSLGTQTYICNVNHKPSNTKVDKKVEPKSCD--  272
32H109L_Fd_cowCDRs       SVVTVPSSSLGTQTYICNVNHKPSNTKVDKKVEPKSCD--  272
PGT145_Fd                SVVTVPSSSLGTQTYICNVNHKPSNTKVDKKVEPKSCD--  244
PGT145_knob_Fd           SVVTVPSSSLGTQTYICNVNHKPSNTKVDKKVEPKSCD--  278
PGT145_Fd_cowCDRs        SVVTVPSSSLGTQTYICNVNHKPSNTKVDKKVEPKSCD--  272
                         *:****.*: * **: *** *  *.***** *******
```

CLUSTAL O(1.2.4) multiple sequence alignment

Underlined residues indicate immunogenic sequences assessed by
http://tools.iedb.org/deimmunization/

```
                               CDR-L1                   CDR-L2
NC-Cow1_LC               -SYELTQPSSVSGSLGQRVSVTCSGSS---SNVGNGYVSWYQLIPGSAPRTIIYGDTSRA 56
trastuzumab_LC           DIQMTQSPSSLSASVGDRVTITCRASQ---D--VNTAVAWYQQKPGKAPKLLIYSASFLY 55
trastuzumab_LC_cowCDRs   DIQMTQSPSSLSASVGDRVTITCSGSS---SNVGNGYVSWYQQKPGKAPKLLIYGDTSRA 57
32H109L_LC               -SYVLTQPSQLSVAPGETARISCGGRS-----LGSRAVQWYQQKPGQAPVLVIYNNQDRP 54
32H109L_LC_cowCDRs       -SYVLTQPSQLSVAPGETARISCSGSS---SNVGNGYVSWYQQKPGQAPVLVIYGDTSRA 56
PGT145_LC                EVVITQSPLFLPVTPGEAASLSCKCSHSLQHSTGANYLAWYLQRPGQTPRLLIHLATHRA 60
PGT145_LC_cowCDRs        EVVITQSPLFLPVTPGEAASLSCSGSS---SNVGNGYVSWYLQRPGQTPRLLIHGDTSRA 57
                               .*  :  : *: . ::*              : **   **.:*  :*:

                                                                CDR-L3
                         _
NC-Cow1_LC               SGVPERFSGSRS---GNTATLTISSLQAEDEADFFCASPDD-SSSNAVFGSGTTLTVLGQ 112
trastuzumab_LC           SGVPSRFSGSRS---GTDFTLTISSLQPEDFATYYCQQHY---TTPPTFGQGTKVEIK-R 108
trastuzumab_LC_cowCDRs   SGVPSRFSGSRS---GTDFTLTISSLQPEDFATYYCASPDD-SSSNAVFGQGTKVEIK-R 112
32H109L_LC               SGIPERFSGSPDSNFGTTATLTISRVEAGDEADYYCHMWDSRSAINWVFGGGTKLTVLGQ 114
32H109L_LC_cowCDRs       SGIPERFSGSPDSNFGTTATLTISRVEAGDEADYYCASPDD-SSSNAVFGGGTKLTVLGQ 115
PGT145_LC                SGVPDRFSGSGS---GTDFTLKISRVESDDVGTYYCMQGL---HSPWTFGQGTKVEIK-R 113
PGT145_LC_cowCDRs        SGVPDRFSGSGS---GTDFTLKISRVESDDVGTYYCASPDD-SSSNAVFGQGTKVEIK-R 112
                         **:*.***** .   *.  **.** ::  * . ::*           .** **.: :  :


NC-Cow1_LC               PKSPPSVTLFPPSTEELNGNKATLVCLISDFYPGSVTVVWKADGSTITRNVE-TTRASKQ 171
trastuzumab_LC           TVAAPSVFIFPPSDEQLKSGTASVVCLLNNFYPREAKVQWKVDNALQSGNSQESVTEQDS 168
trastuzumab_LC_cowCDRs   TVAAPSVFIFPPSDEQLKSGTASVVCLLNNFYPREAKVQWKVDNALQSGNSQESVTEQDS 172
32H109L_LC               PKAAPSVTLFPPSSEELQANKATLVCLISDFYPGAVTVAWKADSSPVKAGVE-TTTPSKQ 173
32H109L_LC_cowCDRs       PKAAPSVTLFPPSSEELQANKATLVCLISDFYPGAVTVAWKADSSPVKAGVE-TTTPSKQ 174
PGT145_LC                TVAAPSVFIFPPSDEQLKSGTASVVCLLNNFYPREAKVQWKVDNALQSGNSQESVTEQDS 173
PGT145_LC_cowCDRs        TVAAPSVFIFPPSDEQLKSGTASVVCLLNNFYPREAKVQWKVDNALQSGNSQESVTEQDS 172
                           : *** :**** *:*:...*::***:.:***  ..* **.*.:  . . : :.  ...

NC-Cow1_LC               SNSKYAASSYLSLTSSDWKSKGSYSCEVTHEGS--TVTKTVKPSECS    216
trastuzumab_LC           KDSTYSLSSTLTLSKADYEKHKVYACEVTHQGLSSPVTKSFNRGEC-    214
trastuzumab_LC_cowCDRs   KDSTYSLSSTLTLSKADYEKHKVYACEVTHQGLSSPVTKSFNRGEC-    218
32H109L_LC               SNNKYAASSYLSLTPEQWKSHKSYSCQVTHEGS--TVEKTVAPTECS    218
32H109L_LC_cowCDRs       SNNKYAASSYLSLTPEQWKSHKSYSCQVTHEGS--TVEKTVAPTECS    219
PGT145_LC                KDSTYSLSSTLTLSKADYEKHKVYACEVTHQGLSSPVTKSFNRGECS    220
PGT145_LC_cowCDRs        KDSTYSLSSTLTLSKADYEKHKVYACEVTHQGLSSPVTKSFNRGEC-    218
                         .:..*: ** *:*:  :::.:  *:*:***:*    * *:.   **
```

Supplementary Table 1

Supplementary Table 1. Primers used for the ligation of Fd and Fc sequences to obtain full-length IgGs

Primer name	Sequence (5' -> 3')
32H109L_Fd_wt_fwd	ctggctagcgtttaaacttaagcttAAGCTTGCCACCATGGATTG
32H109L_Fd_wt_rev	tgtgggtcttGTCGCAGCTCTTGGGTTC
trastuzumab-Fc_fwd	gagctgcgacAAGACCCACACCTGTCCTC
trastuzumab-Fc_rev	tttaaacgggccctctagactcgagCTCGAGTCACTTGCCAGG
