# Supplementary material for: Mechanistic principles of an ultra-long bovine CDR reveal strategies for antibody design
Source: Nat Commun. 2021 Nov 18;12:6737. doi: 10.1038/s41467-021-27103-z (PMC8602281; doi:10.1038/s41467-021-27103-z)
Supplement: Supplementary file 2 — Reporting summary [file 41467_2021_27103_MOESM2_ESM.pdf]

nature portfolio

Corresponding author(s): Buchner, Svilenov

Last updated by author(s): Oct 26, 2021

Reporting Summary

Nature Portfolio wishes to improve the reproducibility of the work that we publish. This form provides structure for consistency and transparency in reporting. For further information on Nature Portfolio policies, see our Editorial Policies and the Editorial Policy Checklist.

Statistics

For all statistical analyses, confirm that the following items are present in the figure legend, table legend, main text, or Methods section.

n/a	Confirmed	
[ ]	[x]	The exact sample size (n) for each experimental group/condition, given as a discrete number and unit of measurement
[ ]	[x]	A statement on whether measurements were taken from distinct samples or whether the same sample was measured repeatedly
[x]	[ ]	The statistical test(s) used AND whether they are one- or two-sided<br>*Only common tests should be described solely by name; describe more complex techniques in the Methods section.*
[x]	[ ]	A description of all covariates tested
[ ]	[x]	A description of any assumptions or corrections, such as tests of normality and adjustment for multiple comparisons
[ ]	[x]	A full description of the statistical parameters including central tendency (e.g. means) or other basic estimates (e.g. regression coefficient) AND variation (e.g. standard deviation) or associated estimates of uncertainty (e.g. confidence intervals)
[x]	[ ]	For null hypothesis testing, the test statistic (e.g. F, t, r) with confidence intervals, effect sizes, degrees of freedom and P value noted<br>*Give P values as exact values whenever suitable.*
[x]	[ ]	For Bayesian analysis, information on the choice of priors and Markov chain Monte Carlo settings
[x]	[ ]	For hierarchical and complex designs, identification of the appropriate level for tests and full reporting of outcomes
[x]	[ ]	Estimates of effect sizes (e.g. Cohen's d, Pearson's r), indicating how they were calculated

Our web collection on statistics for biologists contains articles on many of the points above.

Software and code

Policy information about availability of computer code

Data collection	Astra v5, Blitz software v1.2, Chirascan software v4.5, Jasco manager v2, Microcal PEAQ DSC v1.5, PR. ThermControl v2.1, Biacore X-100 software v2, Amber18
Data analysis	Origin version 2019, GraphPad Prism 9

For manuscripts utilizing custom algorithms or software that are central to the research but not yet described in published literature, software must be made available to editors and reviewers. We strongly encourage code deposition in a community repository (e.g. GitHub). See the Nature Portfolio guidelines for submitting code & software for further information.

Data

Policy information about availability of data

All manuscripts must include a data availability statement. This statement should provide the following information, where applicable:

- Accession codes, unique identifiers, or web links for publicly available datasets
- A description of any restrictions on data availability
- For clinical datasets or third party data, please ensure that the statement adheres to our policy

The manuscript contains a data availability statement. Raw data are available for all figures; there are no deposited data

Field-specific reporting

Please select the one below that is the best fit for your research. If you are not sure, read the appropriate sections before making your selection.

☒ Life sciences ☐ Behavioural & social sciences ☐ Ecological, evolutionary & environmental sciences

For a reference copy of the document with all sections, see nature.com/documents/nr-reporting-summary-flat.pdf

Life sciences study design

All studies must disclose on these points even when the disclosure is negative.

Sample size	typically n was 3. this is the standard number of replicates in biochemical experiments
Data exclusions	no data were excluded
Replication	experiments were typically repeated three times. All replicates are reported in the manuscript and were used to calculate mean values and standard deviations
Randomization	Randomization is not used in biochemical experiments
Blinding	Blinding is not used in biochemical experiments

Reporting for specific materials, systems and methods

We require information from authors about some types of materials, experimental systems and methods used in many studies. Here, indicate whether each material, system or method listed is relevant to your study. If you are not sure if a list item applies to your research, read the appropriate section before selecting a response.

Materials & experimental systems

n/a	Involved in the study	
☐	☒	Antibodies
☐	☒	Eukaryotic cell lines
☒	☐	Palaeontology and archaeology
☒	☐	Animals and other organisms
☒	☐	Human research participants
☒	☐	Clinical data
☒	☐	Dual use research of concern

Methods

n/a	Involved in the study	
☒	☐	ChIP-seq
☒	☐	Flow cytometry
☒	☐	MRI-based neuroimaging

Antibodies

Antibodies used	all antibodies used were produced in the context of this study. No commerical antibodies were used.
Validation	not applicable

Eukaryotic cell lines

Policy information about cell lines

Cell line source(s)	Expi293 cells (ThermoFisher) were used for protein expression, Vero E6 cells (ATCC-CRL-1586) were used for virus neutralization experiments
Authentication	The cell lines were obtained from commercial providers. We did not do further identity tests on the cell line.
Mycoplasma contamination	Not detected.
Commonly misidentified lines (See ICLAC register)	No commonly misidentified cell lines
